# Effects of the delivery of physiotherapy on the treatment course of elderly fallers presenting to the emergency department: Protocol for a randomized clinical trial

**DOI:** 10.1371/journal.pone.0303362

**Published:** 2024-05-08

**Authors:** Marie Blandin, Marie Gallet, Christelle Volteau, Philippe Le Conte, Thomas Rulleau, Guillaume Le Sant

**Affiliations:** 1 Nantes Université, CHU Nantes, Movement – Interactions - Performance, MIP, UR 4334, Nantes, France; 2 IFM3R, School of Physiotherapy, St-Sebastien-sur-Loire, France; University of Campania Luigi Vanvitelli: Universita degli Studi della Campania Luigi Vanvitelli, ITALY

## Abstract

The use of physiotherapy (PT) in the hospital emergency department (ED) has shown positive results including improvements in patient waiting time, treatment initiation, discharge type, patient outcomes, safety and acceptability of the intervention by medical staffs. These findings originate from studies that primarily focus on musculoskeletal and orthopaedic conditions. Despite a significant number of people visiting the ED, there is a shortage of literature evaluating PT in the ED for elderly populations. The objective of this study is the evaluate the effect of delivering PT in the ED (versus no delivery) in patients aged 75 and over with ‘falls’ complaints. The main objective is the evaluate the effect on the discharge disposition (discharge home, hospitalization). Secondarily, we will evaluate the effect delivering PT on patient-length of stay, the number of falls at 7 days after admission to the ED, changes between the initial and final medical decision regarding patient orientation, and medical staff satisfaction. This study will follow a prospective longitudinal design involving participants aged 75 years and over. We plan to recruit a total n = 336 patients admitted to the ED with a ‘fall’ chief complaint. After consent, participants will be randomized into either the ‘PT-group’ (receiving a prescription and execution of PT within the ED), or to the ‘no-PT group’ (no delivery of PT within the ED). The PT intervention will involve a standardized assessment of motor capacities using validated clinical examinations, and the delivery of rehabilitative exercises based on individual needs. Outcomes will be recorded from the patient’s medical record, and a phone call at 7 days. A questionnaire will be sent to medical staff. The results of this study will help to determine whether PT might be beneficial for the management of this increasing proportion of individuals who come to the ED.

**Trial registration:** (Trial registration number: ClinicalTrials.gov NCT05753319). https://classic.clinicaltrials.gov/ct2/show/NCT05753319.

## Introduction

Emergency Departments visits have exponentially growth worldwide in the last decades. The annual number of visits to ED in France has doubled between 1996 (10 million visits) and 2022 (20.5 million) [1], overcrowding ED. This increasing demand raises the risk of suboptimal care delivery. Overcrowded ED have been associated with adverse events on i) patients, including delayed assessment and care, increased levels of morbidity and mortality; ii) carers and staff, leading to higher frequencies of medical errors, exhaustion, stress and reports of violence; iii) health systems, resulting in longer inpatient lengths of stay, increased ED readmissions and associated costs (for more comprehensive reviews, see Morley et al. [2] and Rasouli et al. [3]). These findings are even more pronounced when considering older populations. In 2022, 44.8% of the population aged over 75 years were admitted to a French ED for at least one visit, accounting for 17% of all ED admissions in the country, while people >75 years made up only 9% of the total population [1,4,5]. The duration of ED stay is longer for the elderly (34% of admissions treated in less than 4 hours vs 79% for <18 years, 61% for 18–74 years [6]), resulting in higher risks for adverse outcomes [7,8] and poor management [9–11]. Additionally, almost 50% of ED visits by patients aged 75 years and over are followed by a subsequent hospitalization [1,6].

French authorities have announced a series of measures in response to the ED ‘endless’ crisis. These potential solutions incorporate both outpatient and inpatient care, as by utilizing the skills and competencies of paramedical staff in the ED [12]. Emerging evidence indicates that using physiotherapy (PT) in patient care within ED reduces wait/treatment time, improves patient outcomes, and offers possibilities for discharging medical staff to complex situations without increasing the risk of adverse incidents [13–18]. Physiotherapists are also well-received by patients and ED staff, and their actions are seen as an opportunity to enhance the care provided in ED [16,19]. However, these results mainly stem from studies in which physiotherapists primarily managed musculoskeletal and orthopaedic conditions, with a wide variation in practice patterns (e.g. primary/secondary contact health provider) and their responsibilities.

There is a shortage of literature evaluating the effects of PT in ED for non-musculoskeletal chief complaints [20]. Data from our hospital [21] and from Gurley et al. [22] reported that ‘falls’ in older populations might account for up to 40% of PT prescriptions [21] or interventions [22] within the ED. Another retrospective study also emphasized the potential for delivering PT in the ED to ‘fallers’ [23]. However, this study did not allow for an estimation of the specific nature of PT service provided (e.g. assessment and/or therapeutic sessions; location within the ED units) or the effects produced on patient discharge and length of stay.

The number of patients aged 75 years and over presenting to the ED increasing, so as their primary complaint identified as ‘fall’ [6,24–26]. It is also estimated that more than 50% of fall patients presenting at the ED report adverse events: recurrent falls, revisits to the ED with subsequent hospitalisations within 6–12 months [27,28], and up to 15% mortality rate one year later [28]. Given the challenges in caring for the elderly population due to frailty and their higher risk for adverse outcomes after an ED visit, there is a need for special attention on screening/assessment and strategies offered to this population in ED [11,29]. To the best of our knowledge, no study has prospectively estimated the effect of delivering PT to patients aged 75 and over admitted in ED with a chief complaint of ‘fall’.

Therefore, the main objective of this study is the evaluate the effect of delivering PT in the ED (versus no PT) in patients aged 75 years and over for ‘falls’ complaints, on the discharge disposition after their ED visit.

The secondary objectives are to evaluate the effect of the intervention (PT vs no PT) on:

1. the length of stay (LOS) within the ED and the hospital
2. the number of falls recorded within the 7 days after admission in ED
3. the medical staff satisfaction of PT practice in ED
4. the changes between initial and final EP decision regarding patient orientation (discharge home, acute care unit, hospitalization)

## Materials and methods

### Study design

**Fig 1:**
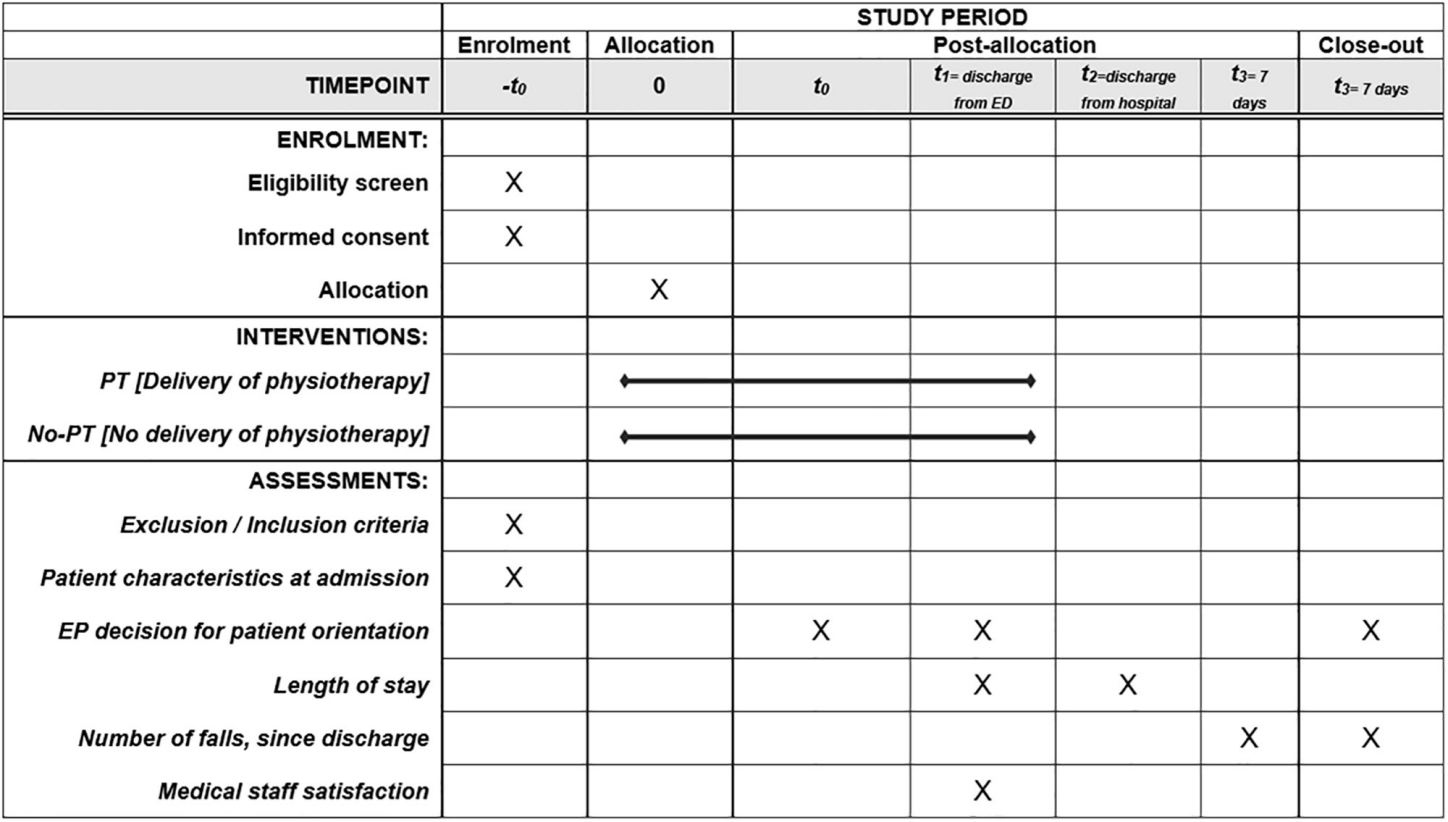
SPIRIT Diagram for the study. Abbreviations: ED: Emergency department; PT: Physiotherapy.

**Fig 2:**
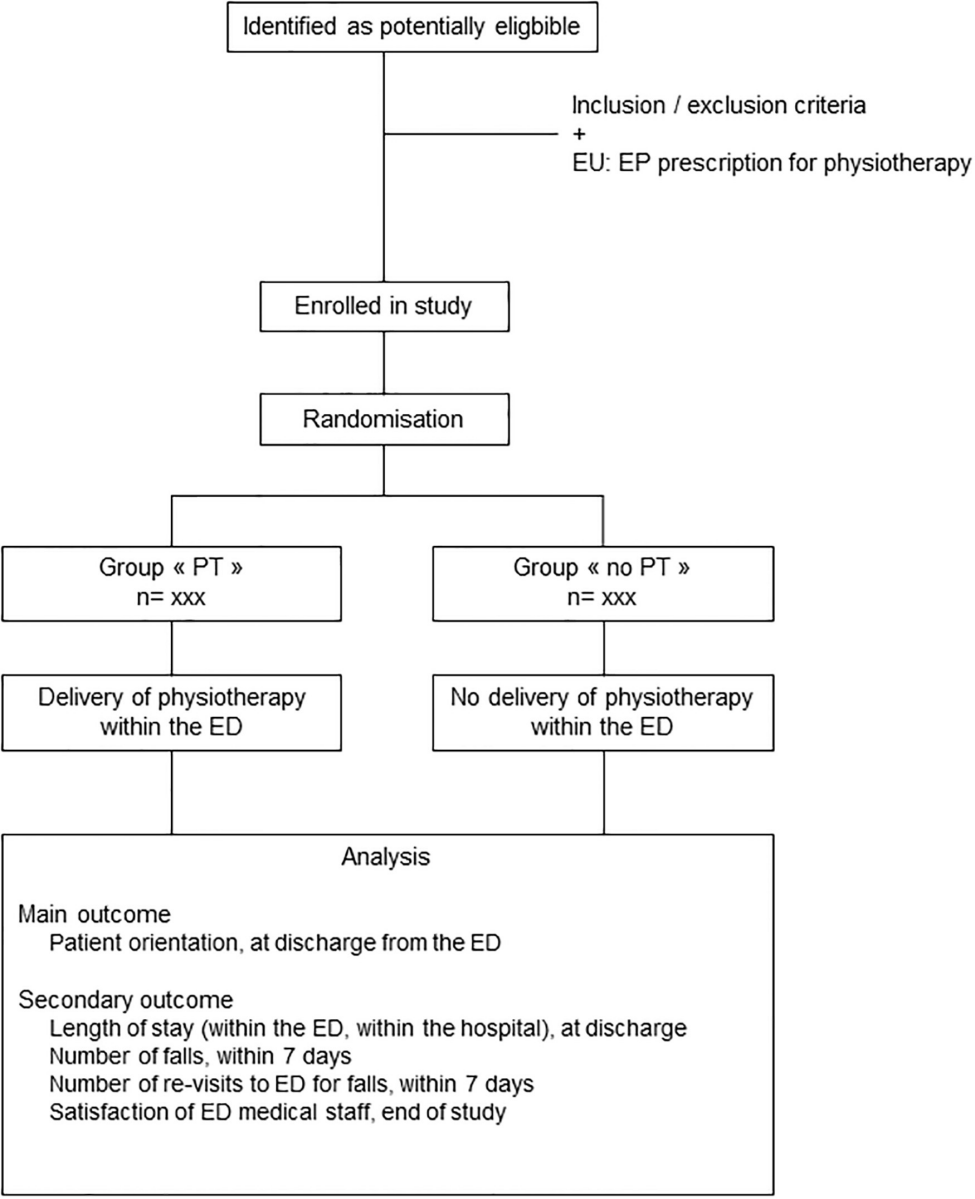
Flowchart of the study. Protocol version: v.2, June 2023. Abbreviations: ED: Emergency department; EP: Emergency physician; EU: Emergency Unit; OU: Observation Unit; PT: Physiotherapy.

This is a prospective open-label, randomized, monocentric, two-arm parallel controlled trial to analyse the effects of the delivery of PT in ED, for patients aged 75 years and over, admitted for ‘fall’ chief complaint. The study is reported according to the Standard Protocol Items: Recommendations for Interventional Trials (SPIRIT) [30]. The SPIRIT diagram and flowchart for participants are provided in Figs 1 and 2, respectively.

The accordance with SPIRIT checklist and a trial registration data set are provided in (S1 and S2 Files, respectively). The exact timetable of the different phases of the study is provided in S3 File.

### Study setting

This study will be conducted in the adult ED of University Hospital (CHU) of Nantes between April 6, 2023 and February 29, 2024. This ED is one the largest in France, with an average of 219 daily visits and a total of 78 908 annuals visits reported in 2021 (adult ED). Among these patients, 14.0% are aged over 75 years (national rate: 13.9%) [6,31].

The ED is divided into different sectors (traumatic, non-traumatic, fast-track, resuscitation room). Following an initial triage by a specialized nurse, patients are directed to one of the aforementioned sectors based on their clinical presentations and severity. Then, at the Emergency Unit (EU), the Emergency Physician (EP) conducts an initial clinical exam leading to: i) an initial course of action, including prescription (medical imaging, clinical investigation/assessments, biological analyses), and/or introduction of treatment; ii) and an initial decision for patient orientation depending on the clinical severity, treatment requirement, and the initial evolution of the patient. This may include decisions such as a hospital admission, discharge with follow-up instructions, or referral to outpatient specialist. Another possibility is to admit the patient to an Observation Unit (OU) that is close to the ED. The OU is dedicated for the surveillance after initial clinical examination of patients with unpredictable evolution. For instance, if the evolution indicates a more severe condition than initially thought, the EP can decide to admit the patient to the hospital instead of sending him home (discharge). Similarly, if the condition significantly improves with the introduction of treatment, the initial decision for hospital admission can be re-evaluated [32].

**Fig 3:**
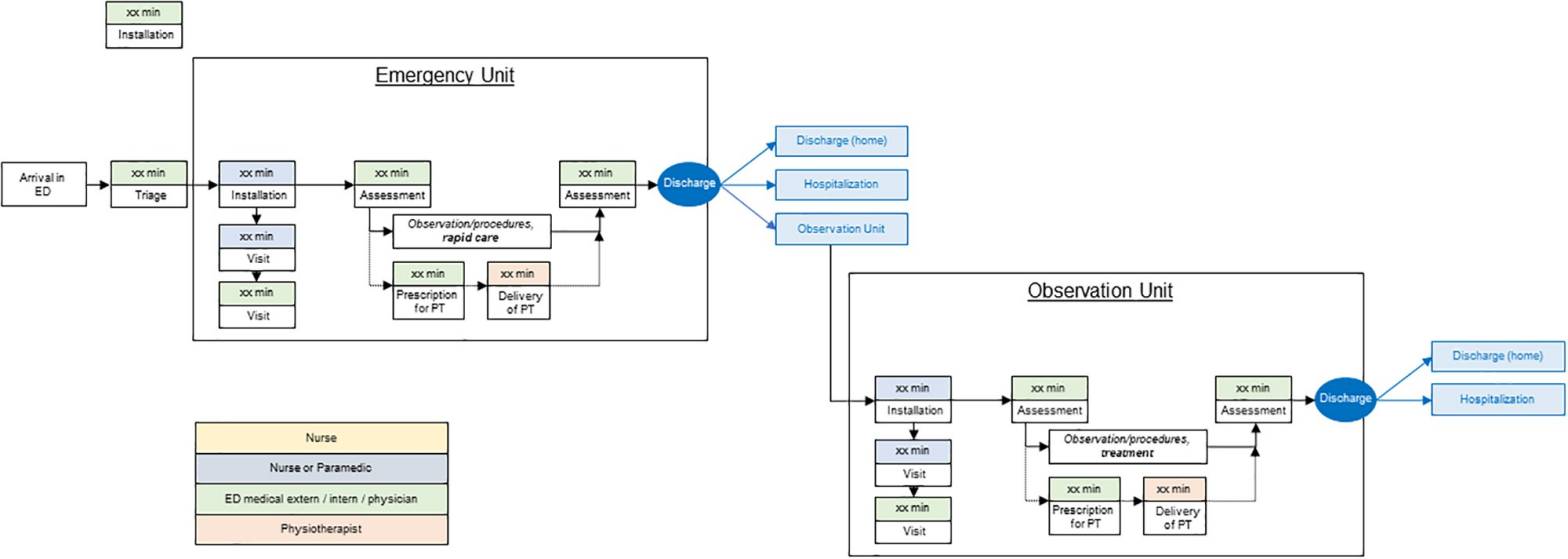
Theoretical mapping of pathway within the ED for a patient ≥75 years presenting for ‘fall’ complaint. Abbreviations: ED: Emergency department; PT: Physiotherapy.

Briefly, patients aged 75 years and over admitted with a chief complaint of a ‘fall’ are first clinically assessed within the EU, to provide a prompt clinical exam, an initial course of action, and an initial decision for orientation. After this first stage, there are three possibilities: (i) the situation is benign and the patient is discharged; (ii) there is a clear indication for hospitalization; or (iii) the clinical situation is unpredictable and the patient can be admitted to the OU for observation/monitoring, treatment, and further assessments. As illustrated in Fig 3, the typical pathway involves patients initially going to the EU where they may be directed to one of the three options: returning home, being hospitalized (in another unit of the hospital), or being transferred to the OU. Once in the OU, these patients may later be directed to either return home or continue hospitalization.

### Recruitment process

The aim is to recruit, as exhaustively as possible, all patients meeting the study’s criteria, over a sufficiently long inclusion period to be representative of the population of elderly patients with falls complaints admitted to the ED of Nantes University Hospital. Preliminary analysis at Nantes University Hospital (in May 2022) suggests that there are around 13 patients/day to be eligible to participate in this study. Based on the daily organization of PT services in the ED and considering approximately that 50% of eligible patient will consent to participate, the aim will be to recruit 4 patients/day. A period of recruitment to last 6 months will be planed for a final recruitment for the trial, that may result for a final recruitment of a total of 336 participants for the trial.

Participants who meet the following criteria will be included:

- Age 75 years and over, at the age of admission
- Admission in ED for ‘fall’ chief complaint
- Able for gait or fall risk assessment
- Able to understand the protocol and not verbally object to participation

Participants will not be included in case of being already included into another research protocol, not providing their informed consent or being placed under guardianship/curatorship. They will also be excluded if they present a fall that may require a surgical treatment, or being diagnosed a systematic pathology (e.g. cancer) within the ED.

### Informed consent

Patients who meet inclusion criteria will be informed of the nature of the study, by the recruiting EP. A description of the trial will be provided to the patients (written and verbal information) and discussions on the information provided, before they give their oral consent to the research investigator. The legal requirement in the country for the study is an oral consent to be given. A third party will act as a witness (Nurse or EP). The oral consent will be recorded by the witness in the medical record.

### Allocation

Participants will be randomly allocated to either study group: PT or no-PT with a ratio 1:1. A SAS program will be used to produce a permuted blocks randomization plan with varying block sizes. This program will be performed by a statistician of the DRI of the University Hospital of Nantes. The randomization will be not stratified.

The investigator can allocate the study group of the included patients using a web browser by connecting to ENNOV Clinical Software: this is a secure, computer-generated, interactive web-response system available at: https://nantes-lrsy.ennov.com/EnnovClinical/login.

### Blinding

It will not be possible to keep physiotherapists of the ED and patients blinded, due to the nature of the study.

### Intervention

Only participants of “PT-group” will receive a delivery of PT within ED, composed by a motor assessment as recommended by French National Guideline [33] and a daily session of rehabilitation until discharge from the ED. The ED physiotherapist will report the results of the examinations and exercises, into the patients’ medical record. In addition, the ED physiotherapist will provide a recommendation regarding the patient’s orientation (discharge/hospitalization). This information will be accessible to the EP to assist in making the final decision regarding patient’s orientation.

#### Motor assessment

A battery of clinical tests are recommended to better estimate balance, motor capacities and risks of falls in older populations, overcoming the psychometric limitations of one test used in isolation [34]. These tests are also chosen in order to be the most suitable in context of emergency medicine, optimizing: the delivery format, the training and time required for administration and the need of assistive devices. The battery of tests lasts 30 minutes to be delivered to a patient aged 75 years and over in context of ED. Before each test, the assessor (ED physiotherapist) will provide instructions and show how to perform the test. One repetition of each test will be used. The Table 1 details the procedures of each test.

**Table 1:** Clinical assessments and exercises used during PT intervention in ED (patient ≥ 75 years).

Name of the Assessment	Description	Threshold for initiating exercises within the ED	Example of exercises
Unipedal Balance Test—UBT [35] Duration: 2 minutes	The patient is asked to stand barefoot on the limb of his choice, with the other limb raised so that the raised foot was near but not touching the ankle of their stance limb. Each patient is asked to focus on a spot on the wall at eye level in front of him/her, for the duration of the eyes open test. Prior to raising the limb, the patient is instructed to cross his arms over the chest. The investigator uses a stopwatch to measure the amount of time the patient was able to stand on one limb. Time starts: when the patient raised the foot off the floor. Time ends: when the patient either: (1) used his arms (ie, uncrossed arms), (2) used the raised foot (moved it toward or away from the standing limb or touched the floor), (3) moved the weight-bearing foot to maintain his balance (ie, rotated foot on the ground), (4) a maximum of 45 seconds had elapsed, or (5) opened eyes on eyes closed trials.	< 5 sec	Unipodal stance exercise (each limb) with eyes opened/closed, for a duration of 6 sec
Romberg test [36] Duration: 2 minutes	The patient stands on the floor, preferably with shoes off, feet together. The patient is asked to maintain the position with eyes open and then closed. The investigator uses a stopwatch to measure the amount of time the patient (upper limit typically = 30 seconds) is able to maintain the position with eyes open then retested with eyes closed. Prior to raising the limb, the patient is instructed to cross his arms over the chest. The Romberg test is positive when the patient is unable to maintain balance with their eyes closed. Losing balance can be defined as increased body sway, placing one foot in the direction of the fall, or even falling.	Positive	Transfer from supine to seated position, seated to bipodal stance
Tinetti Performance Oriented Mobility Assessment Duration: 10 minutes	For testing the balance abilities: The patient is asked to sit, to stay, to rise up and stay standing from an armless chair. Then, the patient is asked to turn 360° and sit back down. The investigator looks at how the patient rises and sits down, stays upright particularly when eyes are closed or when getting pushed against the sternum. For testing the dynamic balance: the patient is asked to walk a few metres (15 feet) at normal speed, to turn and walk back at a speeder (but safe) velocity, and to sit back on the chair. The investigator looks at the quality of the steps (length and height), the symmetry and continuity of the steps, and to the rigidity of the trunk. Brace, cane, walker or any device used to walk can be used during the test. Balance (/16) and gait (/12) scores are summed to 28. The lower the score, the higher the risk of falling.	19	Balance exercise with external perturbation
Time Up and Go Test—TUG [37] Duration: 5 minutes	The patient is sitting in a chair. The patient is asked to stand up (command “go”) and walk forward 3 metres (10 feet), to turn around at the foot mark on the floor and to walk back to the chair. The investigator uses a stopwatch to measure the amount of time between the command to start, till the buttocks touch the chair. Brace, cane, walker or any device used to walk can be used during the test.	> 20 sec	Walking with assistive device related to deficiencies + typical pattern of gait
Stop Walking When Talking test—SWWT [38] Duration: 5 minutes	The patient is walking down in the ED service (in a corridor) under the supervision of the investigator. The supervisor observes whether the patient stops or not walking when a conversation in engaged by the investigator.	Positive	Gait with double task (counting, speaking…)
Ankle range of motion in dorsiflexion Duration: 5 minutes	The patient is supine with knee flexed at 90° and foot in neutral position of inversion/eversion. The investigator stabilizes tibia and fibula to prevent hip/knee compensations. Using a goniometer centered at the bottom center of the lateral malleolus, with proximal arm positioned in reference of fibula head and the distal arm in reference to the 5^th^ metatarsal, respectively, the angulation in maximal passive dorsiflexion angle, is measured at the angle corresponding to the onset of pain	< 5°	Ankle mobilisation/ calf stretches

The One Leg Balance Test (OLBT) also named one-leg balance test or single-balance test will characterize static aspects of standing balance. OLBT is a valid measure for explaining important dimensions in elderly such as frailty, self-sufficiency in activities of daily living, gait performance, and fall status [35,39,40]. For that purpose, the investigator measures the amount of time the patient is able to stand on one limb. We will use the procedures of OLBT described by Springer et al [35]. A duration of a one limb stance <5 sec with eyes opened will be judged as abnormal [33].

The Romberg test will characterize the aspect of balance control due to proprioceptive sensibility [36]. The investigator measures the amount of time the patient is able to maintain balance eyes open or closed. The Romberg test is positive when the patient is unable to maintain balance with their eyes closed. Losing balance can be defined as increased body sway, placing one foot in the direction of the fall, or even falling.

The Tinetti Performance Oriented Mobility Assessment (TPOMA) will be used to characterize balance and gaits impairments during daily activities for elderly populations [41]. The test is composed by two short sections examining the static balance abilities in a chair and standing, and the gait capacities.

The Time Up and Go test (TUG) will be used to assess mobility, balance, and walking ability [37]. TUG may also assist clinicians to identify patients at risk of falling. [42] The investigator measures the amount of time it takes for a patient to stand up, walk 10 ft, walk back and sit down to the same chair. The faster the test, the lower the risk of falling. A TUG>20 sec is associated as a high risk of falling.

The Stop Walking When Talking test (SWWT) will be used to assess the ability to successfully dual task walking with talking. This test examines the relationship between changes in gait and attention-demanding task performance. The SWWT test is also used for fall predication in elderly populations [43]. The test is positive when the patient stops walking when engaged in conversation.

Ankle flexibility in dorsiflexion will be measured through a manual examination of ankle joint range of motion in dorsiflexion, due to the necessity of adequate range motion for executing balance strategies in order to prevent a fall [44].

#### Rehabilitation exercises

A rehabilitation session will be delivered after the clinical assessment, and will last 30 minutes. Different types of exercises will be used, including verticalization exercises, mobility/balance work (eyes open/closed), gait exercises (with or without an assistance device), patient education, tailored to each patient’s needs. The types of exercises are described in Table 1. Note that rehabilitation exercises will be provided/adjusted to patient’s condition each day, until the discharge of ED unit.

### Outcomes

All outcomes, measurement time points and aggregations methods are described in Table 2.

**Table 2:** Summary of outcomes.

Variable	Aggregation method	
	‘PT-group’	‘no PT-group’
Age, at ED admission	mean (SD)	
Gender		
female	n (%)	
male	n (%)	
Emergency Severity Index Level		
1	n (%)	
2	n (%)	
3	n (%	
4	n (%)	
5	n (%)	
Average	mean (SD)	
Primary diagnosis		
Toxicologic	n (%)	
Traumatic	n (%)	
…	n (%)	
Orientation, at discharge from ED (EU patients)		
Return to home	n (%)	
Hospitalization	n (%)	
SSHU	n (%)	
Orientation, at discharge from ED (OU patients)		
Return to home	n (%)	
Hospitalization	n (%)	
Number of falls, within 7 days	n (%)	
Average	mean (SD)	
Number of re-visits to ED for falls, within 7 days	n (%)	
Average	mean (SD)	

Abbreviations: ED: Emergency department; EU: Emergency Unit; OU: Observation Unit; PT: physiotherapy; SD: Standard deviation.

Characteristics of patients at admission to ED will include: gender and age, emergency severity index level, and primary diagnosis.

The primary outcome will be the patient orientation at discharge from the ED according to the defined pathways: return to home (after ERU or SSHU), hospitalization (after ERU or SSHU) or SSHU (after ERU).

The secondary outcomes will be: the length of stay at the ED, or hospital (LOS-ED, LOS-hos) calculated as the duration between admission and discharge from the ED and hospital; the number of re-visits to ED for fall complaints (within 7 days). These outcomes will be found from the medical record of the patient. In addition, a study investigator will call the patient (phone) after 7 days to collect the number of falls since discharge from ED (follow-up).

**Fig 4:**
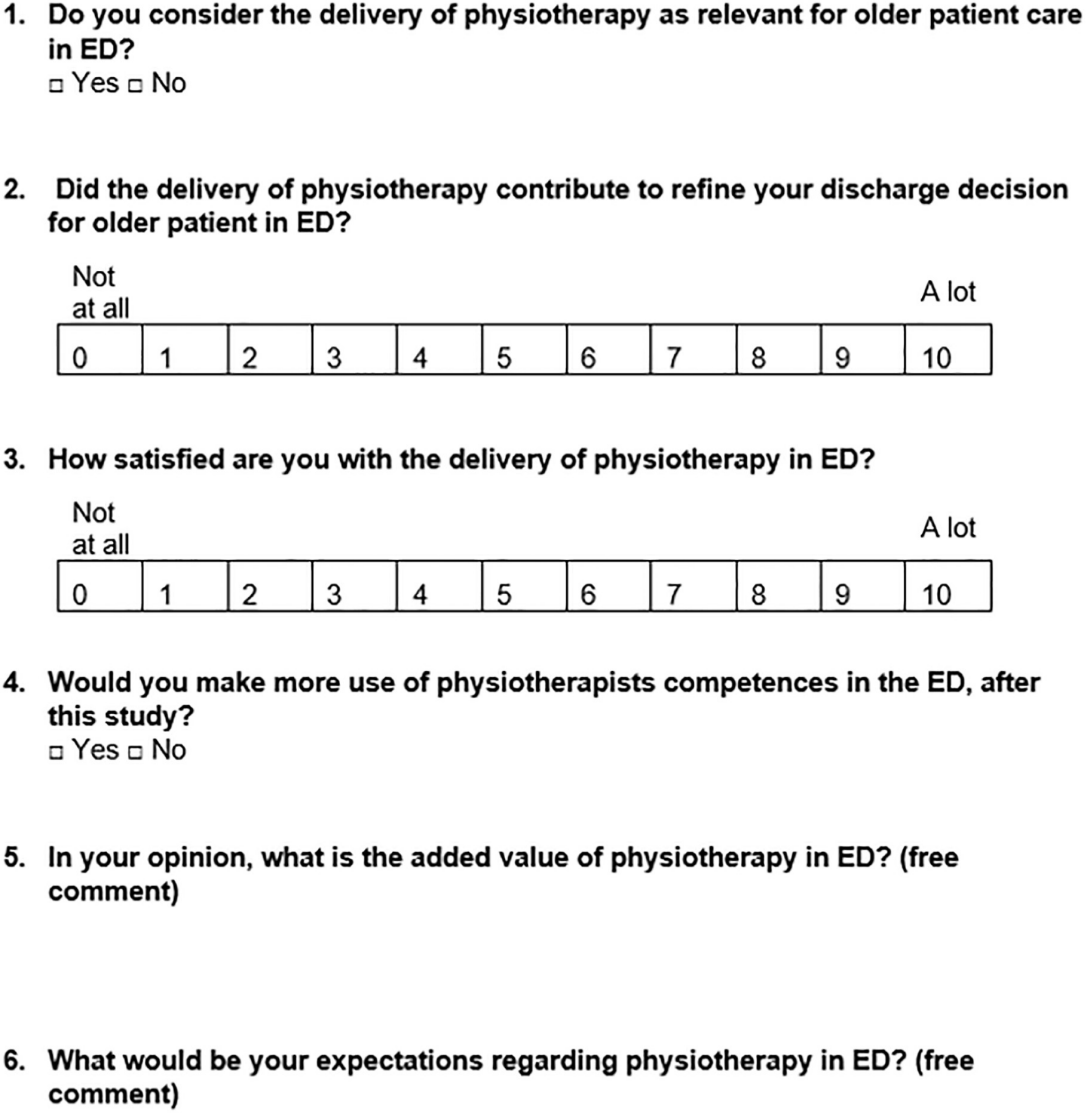
Satisfaction-questionnaire for medical staff.

At the end of the study, the ED medical staff will be asked about their experiences in providing PT within ED: for elderly populations, and in general context. To gather this information, a satisfaction questionnaire containing 4 closed-ended questions, and 2 open-ended questions (for general comments) will be sent electronically via email (Fig 4).

### Data analysis and interpretation

An independent statistician will perform will perform the analyses (SAS, 9.4^®^).

A review of data will be conducted at the end of the study, prior to statistical analysis. The aim will be to review the progress of the study, potential problems and classify any minor or major deviations. Analyses will be performed in the *Intention-to-Treat* populations corresponding to all randomized patients. A sensitivity analysis will be conducted on the *Per-Protocol* population. This population will include patients without major deviation.

The variables measured at baseline will be described for all patients in both groups by numbers and percentages for each category for categorical variables and the minimum, maximum, average, standard deviation and quartiles for the quantitative variables. (Table 2).

Primary endpoint: the patient orientation at discharge from the ED will be described and compared between the two groups with Chi-square tests, depending on the ED unit of inclusion (emergency unit: 3 modalities: discharge home, hospitalization, observation unit; observation unit: 2 modalities: discharge home, hospitalization).’

Secondary endpoints: LOS-ED and LOS-hos will be described as mean difference and 95% confidence intervals, and compared between the groups using Student’s t tests. The rate of re-visits to ED for fall complaints (characterized as a new episode of fall whose severity requires a re-hospitalization within 7 days) will be compared between groups using Chi-square test. The changes in the patient’s orientation between initial and final decisions will be compared between the groups using Chi-square test. Satisfaction of ED medical staff will be depicted descriptively.

Due to this ‘pragmatic’ approach, it is not possible to compute a priori statistical calculations. The distribution of patients within both units of the ED (EU or UN), the different modalities of patient discharge (Fig 3), and for each group (PT vs no-PT) make it challenging to determine expected differences between groups for the main outcome. As stated, we aim to recruit, as exhaustively as possible, during the inclusion period.

Any missing data will be described and explained. There will be no imputation done on missing data.

### Ethical considerations and data management

This study and all of its components were approved by local Ethics committee (GNEDS, n° 23-30-02-230, date: February 23, 2023). The protocol of the trial was also registered with the US National Institute of Health database (NCT05753319). The study protocol approved by the Ethics committee is provided in S4 File (english version and original version).

Data will be logged in anonymous data sheets, accessible and stored electronically on a password-protected software (ENNOV Clinical). Only researchers will be allowed to access to the data. The software enables the identification of individuals who logged in and their actions, so as to restore different document versions. Data will be stored Nantes hospital-university centre for 15 years after completion of the study. Coded data and statistical scripts will also be stored for archiving purposes.

Individual data will not be shared (or only without identification, i.e. coded) for primary and secondary outcomes in publication, in an open archive repository (HAL). According to the European Union General Data Protection Regulation and French laws, each participant will be aware on their rights: to access, rectify and erase their personal data, so as the right to data portability. We do not plan to transfer data outside the EU countries.

## Discussion

### Originality and performance of the study

The results of this study will address existing gaps in the literature, by investigating the effects of PT in ED settings, with a particular focus on falls complaints. We aim to provide comprehensive answers to better understand the extent to which the delivery of PT might improve patient outcomes for elderly populations, who are major users of ED.

Moreover, the study will focus on the perceptions of the medical staff and identify barriers and facilitators to PT within ED. These results will also provide results favouring the dissemination of the results and influence current practices.

### Issues related to recruitment

The use of ED is very popular by populations aged 75 years and over for fall complaints. The inclusion criteria aim to facilitate the recruitment within ED, for this study. We anticipate our recruitment flow to be relatively smooth. Any recommendations to the DMC might be made to modify recruitment if judged necessary. The recruitment status will be reviewed every 2 months to determine the likelihood of meeting recruitment timeline. If recruitment is slower than expected, the recruitment period will be extended until recruitment is successfully concluded.

### Issues related to trial design

This study uses a pragmatic approach aimed at reflecting and informing clinical practice.

Elderly populations were not involved as patient-partners. The objectives and outcomes of this research were defined according to the literature and clinical expertise among investigators. The study design also took into consideration the practical aspects of ED, and its inherent challenges, including professional overuse. Given these circumstances, it seemed reasonable to structure the trial in a way that seamlessly integrates into the regular functioning of the ED.

We should carefully ensure that the study might not disrupt or interfere with standard patient care, and aligns with the day-to-day operations of the ED. Our approach aimed to capture the authentic conditions and demands of the ED environment while conducting the trial. In this specific setting, it would appear that the measurements other risk factors for falls (e.g., muscular performance, sensory disturbances, reduced visual acuity, [45]), or the use of standardized batteries (e.g. BESTest, which requires time/equipment for administration) could not be measured in a pragmatic study of the almost usual care in the ED. Information about patients’ medications is also incomplete when patients arrive at the ED, and would appear to be challenging to collect in the present study.

### Issues related to adverse events

A serious adverse event related to this study would be a fall during the delivery of PT. Investigators may report any adverse event (with or without an expected relationship with the intervention) to the study coordinator. If so, a report to the data monitoring committee (DMC) will be made by the investigators. Appropriated care within the ED will be provided in case of adverse event and for post-trial care, if judged necessary. It Is within the scope of the DMC to communicate to relevant parties (e.g., investigators, Ethics committee) if necessary.

### Monitoring and auditing

The study was designed by clinicians and researchers from complementary fields: emergency medicine, rehabilitation sciences, and biostatistics, from the ED, the Hospital, and the School of Medicine. These members will compose the DMC. Each two month, the role of the DMC will be to examine the flow of recruitment, examine potential adverse events, and make recommendations to protect participants’ safety and protocol modifications (e.g., changes in eligibility criteria, outcomes or planned analyses). The analyses will be performed by the trial statistician. An external auditing will be made at mid-recruitment and at the estimated date of study completion, involving peers’ researchers from the Hospital and the University, familiar with the ED, but not affiliated with the project. This audit will confirm that the regulatory binder is complete and that all associated documents are up to date.

### Dissemination

The results of this study will be disseminated to through our and neighbouring EDs, and through professional emergency and physiotherapy networks and faculties (e.g., lectures). We will also apply to present the trial results at conferences among these fields. Scientific written reports of the findings will conform to the Consolidated Standards of Reporting Trials (CONSORT) statement guidelines. The publication authorship will be determined based on the International Committee of Medical Journal Editors (ICMJE) guidelines. We intend to publish in a reputable non-predatory journal, selecting the most relevant one in using emergency and/or physiotherapy related-categories based on SCImago Journal ranking. We may also considerate the necessity of engaging a professional medical writer.

## Supporting information

S1 File SPIRIT 2013 checklist. (DOC)

S2 File Trial registration data set. (DOCX)

S3 File Timetable of the different phases of the study. (PDF)

S4 File Study protocol. (PDF)
