# Supplementary material for: Effects of the delivery of physiotherapy on the treatment course of elderly fallers presenting to the emergency department: Protocol for a randomized clinical trial
Source: PLoS One. 2024 May 8;19(5):e0303362. doi: 10.1371/journal.pone.0303362 (PMC11078381; doi:10.1371/journal.pone.0303362)
Supplement: S2 File — (DOCX) [file pone.0303362.s002.docx]

**Supporting Information file S2**

**Items from the World Health Organization Trial Registration Data Set**

| **Data category** | **Information** |
| --- | --- |
| Primary registry and trial identifying number | ClinicalTrials.gov - NCT05753319 |
| Date of registration in primary registry | 10 Feb, 2023 |
| Secondary identifying numbers | RC23_0068 |
| Source(s) of monetary or material support | CHU Nantes, Appel d’offres interne Paramedical, 2022 |
| Primary sponsor | CHU Nantes |
| Secondary sponsor(s) | - |
| Contact for public queries | thomas.rulleau@univ-nantes.fr |
| Contact for scientific queries | guillaume.le-sant@univ-nantes.fr |
| Public title | Physiotherapy in emergency department for elderly fallers (KiUrge) |
| Scientific title | Effects of the delivery of physiotherapy on the treatment course of elderly fallers presenting to the emergency department: protocol for a randomized clinical trial |
| Countries of recruitment | France |
| Health condition(s) or problem(s) studied | Fall, elderly |
| Intervention(s) | Experimental: delivery of physiotherapy within the emergency department Comparator: no delivery of physiotherapy |
| Key inclusion and exclusion criteria | Ages eligible for study: ≥75 years Sexes eligible for study: both Accepts healthy volunteers: no Inclusion criteria: adult patient (≥ 75 years), admitted for fall in the ED Exclusion criteria: inclusion into another research, not providing consent, under guardianship/curatorship, if presenting a fall that may require a surgical treatment, or being diagnosed a systematic pathology (e.g. cancer) within the ED. |
| Study type | Allocation: randomized Intervention model: parallel assignment Masking: open-label  Primary purpose: therapy Phase II |
| Date of first enrolment | June 2023 |
| Target sample size | 336 |
| Recruitment status | Recruiting |
| Primary outcome(s) | Discharge disposition after their ED visit |
| Key secondary outcomes | the length of stay (LOS) within the ED and the hospital  the number of falls recorded within the 7 days after admission in ED  the medical staff satisfaction of PT practice in ED  the changes between initial and final physician decision regarding patient orientation (discharge home, acute care unit, hospitalization) |
