# Supplementary material for: Effects of the delivery of physiotherapy on the treatment course of elderly fallers presenting to the emergency department: Protocol for a randomized clinical trial
Source: PLoS One. 2024 May 8;19(5):e0303362. doi: 10.1371/journal.pone.0303362 (PMC11078381; doi:10.1371/journal.pone.0303362)

Study flow chart

Phase			Stage date phase start	Phase duration (in days)	Stage End of phase	Stage date phase end
**Step 1**	Research project instruction	**Success in the AAP and notification of credits**	12/12/2022	73	**Ethical advice**	23/02/2023
**Step 2**	Period of inclusion and/or data collection	**Ethical advice**	23/02/2023	432	**End of the inclusion period**	30/04/2024
**Step 3**	Period of inclusion and/or data collection	**End of the inclusion period**	30/04/2024	185	**Base gel**	01/11/2024
**Step 4**	Data analysis	**Base gel**	01/11/2024	187	**Final report**	07/05/2025
**Step 5**	Valorization	**Final report**	07/05/2025	117	**Publication submission**	01/06/2025
		**Publication submission**	01/06/2025	92	**Publication princeps**	01/09/2025

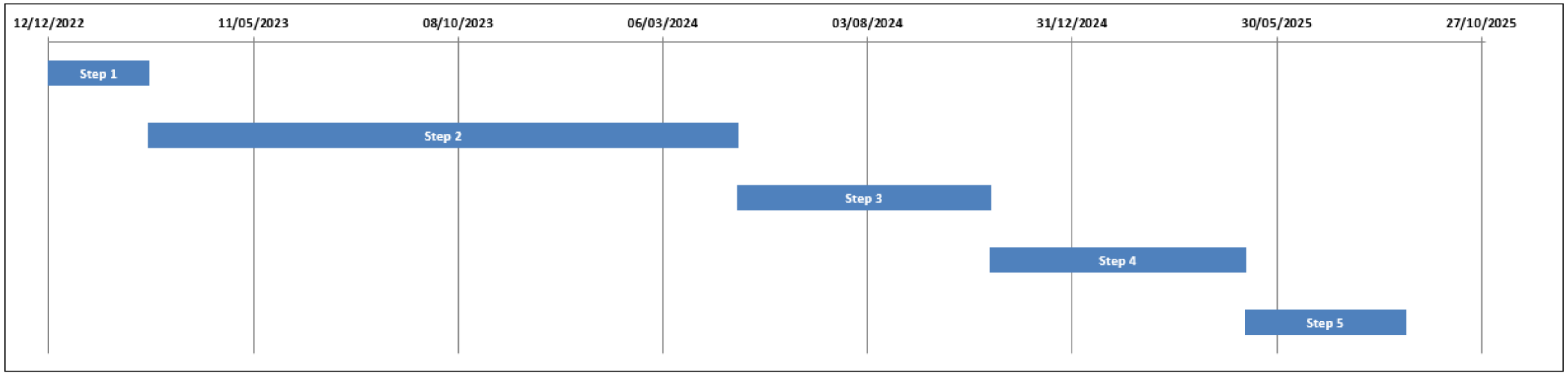

Supplement: S3 File — (PDF) [file pone.0303362.s003.pdf]
