# Supplementary material for: Effects of the delivery of physiotherapy on the treatment course of elderly fallers presenting to the emergency department: Protocol for a randomized clinical trial
Source: PLoS One. 2024 May 8;19(5):e0303362. doi: 10.1371/journal.pone.0303362 (PMC11078381; doi:10.1371/journal.pone.0303362)
Supplement: S4 File — (PDF) [file pone.0303362.s004.pdf]

Research title: masseur-KInésithérapeute en service d'URGEnces : quel(s) impact(s) a l'intervention du MK sur le parcours de soins de la personne âgée chuteuse ?

Research acronym: KiUrge

Reference: RC23_0068

Developer:

Nantes University Hospital
Research and Innovation Department
5, allée de l'île Gloriette
44 093 Nantes cedex 01 (FRANCE)

Bp-prom-regl@chu-nantes.fr
Tel: 02 53 48 28 35
Fax: 02 53 48 28 36

Project sponsor / Coordinating investigator
Marie BLANDIN
Masseur-kinésithérapeute D.E., CHU de Nantes - Hôtel Dieu
marie.blandin@chu-nantes.fr

Associate investigator
Marie GALLET (Masseur-kinésithérapeute D.E., CHU de Nantes - Hôtel Dieu)

Employees
Guillaume LE SANT (PhD PT, University of Nantes)
Thomas RULLEAU (PT PhD, Paramedical research coordinator, CHU Nantes) Christelle VOLTEAU (Methodologist and biostatistician, CHU Nantes)

- The purpose of research is to develop biological or medical knowledge:

  yes ☐ no ☒

- The research is carried out under the direction and supervision of a physician or a qualified person with appropriate experience: yes ☒ no ☐

Index of abbreviations

BDK: Bilan-diagnostic kinésithérapique

MAG: Médecine Aigue Gériatrique

MK: Physiotherapist

MPG: Médecine Polyvalente Gériatrique

MPU: Médecine Polyvalente d'Urgence

RAD: Retour à Domicile

SAU: Service d'Accueil des Urgences (Emergency Department)

UHCD: Unité d'Hospitalisation de Courte Durée (Short-term hospitalization unit)

1. Research interest and relevance

a) Background and rationale

The over-subscription of emergency services is a major problem for the French healthcare system. These services are at the heart of the system's difficulties in meeting people's healthcare needs. In 2019, more than 22 million emergency room visits were recorded in the 697 emergency departments (public and private), a figure that is constantly rising:
+15% compared to 2012, while "only" 10 million passages were observed twenty years earlier in 1996 (Mesnier & Carli, 2019) (DRESS, 2021). This growth creates a tension induced by this over-solicitation, which increases the risk of degrading the quality of care provided to patients and on the activity of caregivers in emergency departments. In order to find a solution to this problem, the Pacte de Refondation des urgences (Report
The "Carli-Mesnier" Report, initiated in 2019, and the recent Flash mission on emergencies and unscheduled care ("Braun" Report) in 2022, propose a number of measures upstream, downstream and within emergency departments. In particular, the "Carli-Mesnier" Report proposes to
"Strengthen and recognize the skills of emergency professionals" (Measure 8).

In some Anglo-Saxon countries, masseur-physiotherapists (MKs) have been treating patients admitted to emergency departments for minor traumatic problems since the 1970s. Orthopedic reasons (sprains, fractures...) or rheumatology (persistent low-back pain, arthrosis...) are the main situations for which MKs are called in (Stanhope et al., 2012). MKs can play 2 distinct roles: an assessment and/or treatment role following a physician referral, or a primary care role that includes direct patient access, treatment plan development and patient discharge (Desmeules et al., 2012; Matifat et al., 2019). The results of these interventions report in the literature: an improvement in direct and indirect costs related to the health situation (e.g. hospitalization duration and services, out-of-hospital consumption), a maintained safety of care (when the MK intervenes in direct access), and a significant degree of team/patient satisfaction (Lafrance, S et al.,2021Matifat, E et al.,2019; Fruth, SJ & Wiley, S.,2016; Morris, J et al.,2015; Taylor, N et al.,2011;Stanhope, J et al.,2012). We can wonder about other areas where physiotherapy could be an interesting recourse. In addition to these studies on physiotherapy in orthopedics and rheumatology, Lefmann and Sheppard (2014) report that physiotherapy could be of interest in other fields of activity, in emergency departments: in particular in gerontology (e.g. for problems linked to falls, balance), or neurology (e.g. for swallowing disorders...) (Lefmann & Sheppard, 2014).

Medical prescriptions in Nantes University Hospital's emergency services point in this direction, as MKs are regularly prescribed procedures related to gerontology, swallowing and vertigo. The prospect of using MKs' skills with elderly populations in emergency departments appears to be a solution worth exploring to manage the issues outlined above. This is all the more important in view of the population seen in emergency departments at Nantes University Hospital. In fact, a survey carried out in May 2022 on Nantes CHU's emergency departments shows that :
- only 20% of patients came to the emergency department for reasons related to benign traumatology (a patient population corresponding to expectations identified in Anglo-Saxon literature).
- 42% were elderly patients hospitalized for falls, representing an average of 13 patients per day on the ward.

Patients aged 75 and over make up a special group of emergency department patients for a number of reasons. Frailty, dependency and polypathologies, which are more frequent among these patients, warrant more complex care than for other patients and lead to longer lengths of stay (Boisguérin & Mauro, 2017). Emergency department admission is a major risk factor for deterioration in the elderly: 23% of patients aged 75 and over spend more than 8 hours in the emergency department, and are much more prone to the occurrence of adverse events during their stay (Mesnier & Carli, 2019). For these patients, the department's MKs are currently asked to carry out autonomy and falls risk assessments. The prescriptions aim to develop an optimal care pathway for these patients. These assessments complement the initial medical diagnosis, and are intended to provide physicians with new arguments for their discharge decisions. Following admission to the Emergency Department (SAU), three medical discharge decisions are possible for these patients: a return home (RAD), hospitalization in the Short-Term Hospitalization Unit (UHCD), which must not exceed 48 hours, or longer hospitalization in one of the geriatric services (Acute Geriatric Medicine (MAG), Multi-Purpose Emergency Medicine (MPU), Multi-Purpose Geriatric Medicine (MPG), Continuing Care and Rehabilitation....) (see Figure 1).

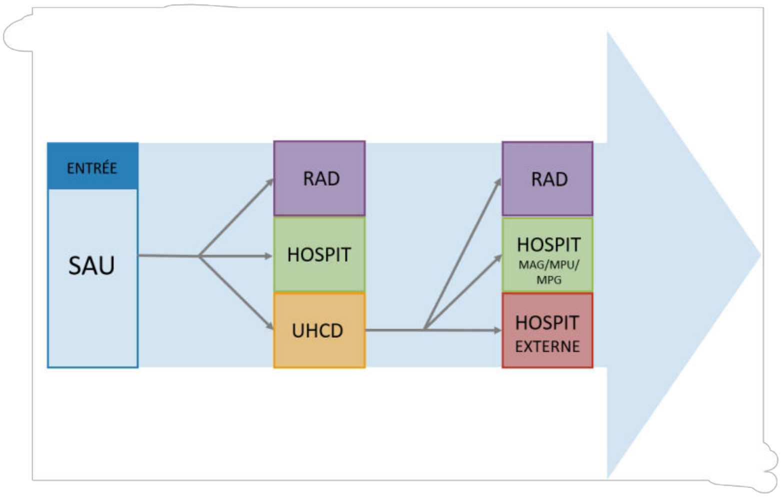

Figure 1- Possible care pathways for elderly patients admitted to the SAU and UHCD (SAU: Services d'Accueil des Urgences, RAD: Retour A Domicile, UHCD: Unité d'Hospitalisation de Courte Durée)

The initial physiotherapy diagnostic workup (BDK) can play a role in guiding the care pathway.

Based on data from research into traumatology in emergency departments, adapted to the context of Nantes, where the percentage of emergency room visits by patients aged 75 and over for falls is high, we question the impact of the MK's action (BDK +/- session) on the patient's pathway.

The initial BDK can be performed at two points in time:

- UAA
- At the UHCD, when patients are transferred there quickly after their admission to the SAU.

Our aim is to assess the impact of BDK, whether performed in the PACU or UHCD, on the medical decision to discharge.

b) Primary objective and primary endpoint

The main aim of this study is to compare patient referral at discharge from emergency departments between patients receiving an MK diagnostic work-up and patients without an MK work-up.

The **main criterion** was patient orientation at discharge from the PACU and UHCD, according to the pathways defined downstream (ARD/UHCD/hospitalization for PACU inclusions or ARD/hospitalization for UHCD inclusions).

c) Secondary objectives and secondary endpoints

<u>Secondary objectives:</u>

1. Compare the length of stay in emergency facilities between the two groups of patients (benefiting from MK vs. not benefiting from MK).
2. Compare overall hospital length of stay between the two patient groups.
3. Compare the rate of recurrence of falls between the 2 groups.
4. Evaluate the satisfaction of physicians working in the UAS and UHCD with the contribution of physiotherapy to patient care.
5. In the group of patients with MK assessment, compare the medical referral decision at discharge from the UAS and UHCD, before and after MK assessment.

<u>Secondary endpoints:</u>

1. Length of stay in UAS and UHCD: calculation of length of stay in hours by recording dates and times of admission to and discharge from the department.
2. Total duration of hospitalization for each stay, all departments combined: calculation of duration in hours by recording dates and times of admission to the emergency department and final discharge from the hospital.
3. Recurrence of admission to an emergency department, for the same reason, of the elderly person within 7 days of returning home. A telephone call will be made to the patient or family 7 days after discharge. The questions asked will be: "Have you relapsed since returning home? If so, how many times? Have you been admitted to the emergency department for this reason?
4. UAS physician satisfaction. To assess this satisfaction, a standardized questionnaire will be sent to all the doctors involved in the care of the study patients (Appendix 2).
5. Referral on discharge from the UAS and UHCD before and after assessment by the MK.

2. Study organization

a) Study diagram

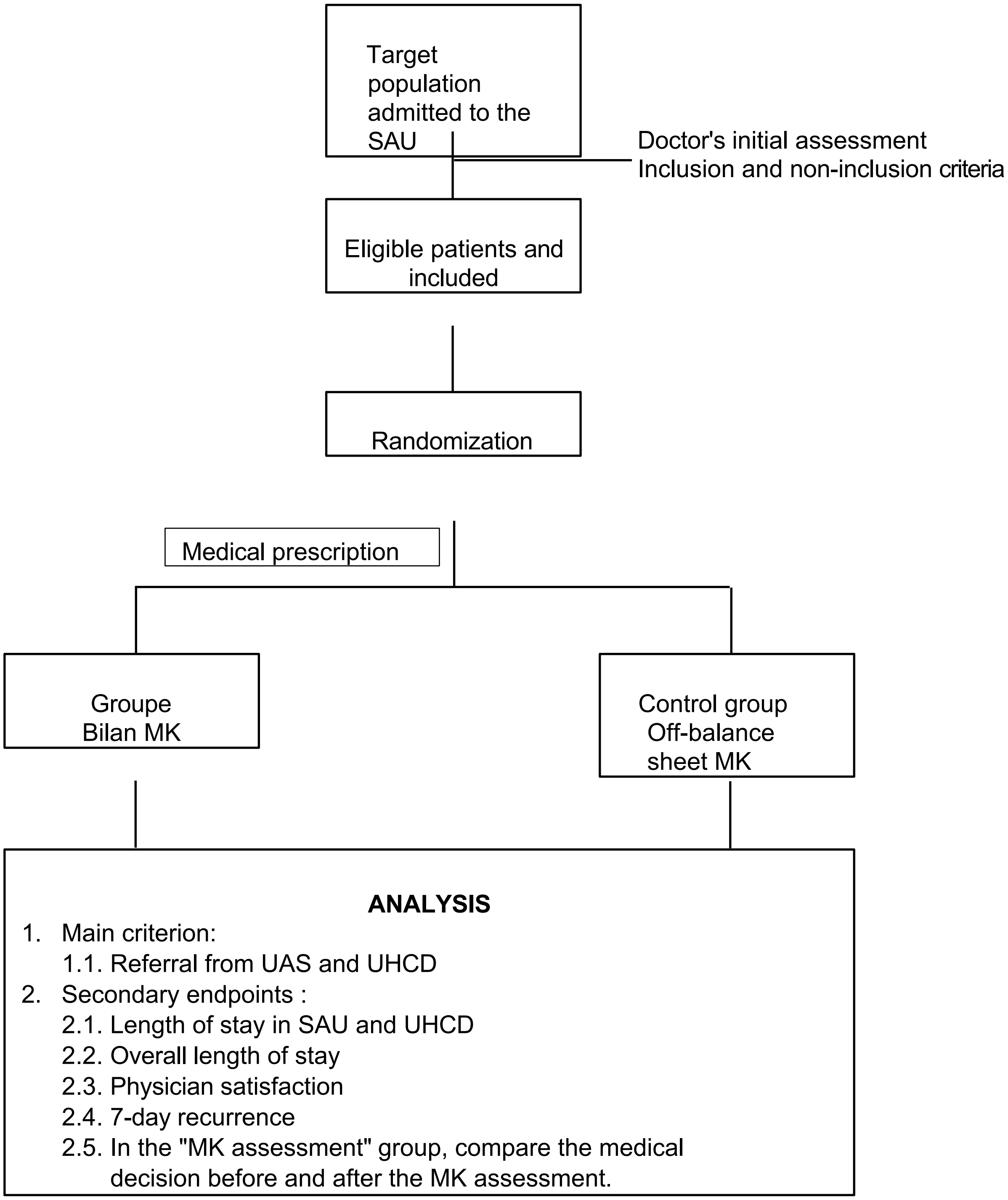

Bilan MK description:

According to the 2009 HAS recommendations (latest recommendations online), the following procedures and techniques will be used during the check-up:

Act/Procedure	Technical / conditions	Frequency	Duration
**MK balance sheet**	Tinetti test	1 time	10 min.
	Time Up and Go test	1 time	10 min.
	Unipodal test	1 time	2 min
	Romberg	1 time	2 min
	Stop walking when talking test	1 time	1 min
	Joint assessment MI	1 time	5 min
**Session se ssion**	Depending on the balance sheet	Every hospital day sation	30 min.

b) Method

The main features of the research are as follows:

- Single-center pilot study not covered by the Jardé law.

  Our position outside the Jardé law is motivated by the fact that research does not aim to develop biological or medical knowledge, and therefore does not include the collection of health data in patient evaluation criteria.

- Prospective, randomized, controlled study (1:1 ratio): patients with systematic MK assessment after medical consultation versus patients without MK assessment.

After this short-term monocentric pilot study, which aims to ensure the feasibility of the protocol and collect data, we want to enable a larger-scale study questioning the value of emergency room physiotherapy at national level and in the medium/long term on the patient pathway and its medico-economic impact.

Course of the study

The aim of this pilot study is to evaluate the effects of an MK diagnostic assessment after the medical consultation on arrival at the emergency department. The study's main criterion is patient referral on discharge from the UAS and UHCD.

A computer alert will be set up on FirstNet, so that the TEC can screen eligible patients. FirstNet is a software program that provides a list of patients admitted to the SAU, by sector. A medical and paramedical team is assigned to each patient.

Inclusion may take place in the PACU, or possibly in the UHCD when patients are transferred there before they can be included and assessed in the PACU. For example, a patient admitted to the PACU at 6pm and transferred to the UHCD during the night could be included and randomized only in the UHCD.

For all eligible patients, it is necessary to check with the medical referent that the patient is clinically stable and able to benefit from a gait assessment or falls risk assessment.

Randomization will be carried out by the TEC. The physician will write a prescription for patients included in the "Bilan MK" group.

If a patient is not included, the doctor who wishes to have a BDK can prescribe it, and it will be carried out according to current possibilities (outside the study).

In order to reduce bias related to the weekly organization of departments, patients entering the emergency department from Monday morning to Thursday lunchtime will be included. Indeed, lengths of stay may be influenced by admissions on other days of the week, particularly weekends (fewer staff, fewer inter-department transfers, etc.). Also, at weekends, only respiratory physiotherapy is carried out, so the MK on duty is unable to carry out assessments in connection with the project.

c) Workforce (justification, assumptions, sampling, depending on the nature of the study)

There are no data in the literature to justify the number of patients to be included in the study. The aim is therefore to recruit, as exhaustively as possible, all patients meeting the study's inclusion and non-inclusion criteria, over a sufficiently long inclusion period (6 months) to be representative of the population of elderly patients with falls admitted to the emergency department of Nantes University Hospital. Preliminary analysis of emergency room visits at Nantes University Hospital (in May 2022) suggests that there are around 13 elderly patients with falls on a daily basis.

Over 6 months of inclusion, the theoretical inclusion capacity for this population would be around 1092 patients (13 patients x 3.5 days x 24 weeks = 1092 patients).

At present, 20% of physiotherapy time is allocated to emergency departments, representing around 3 patients seen per day.

In order to continue with existing treatments (respiratory physiotherapy, swallowing assessments, etc.), while enabling the study to run smoothly, we plan to finance 30% additional MK time. This will enable us to include 4 patients per day, i.e. **336 patients** (4 patients x 3.5 days x 24 weeks = 336 patients).

b) Study population, subjects studied

The population concerned by this study is that aged 75 and over admitted to the SAU for the reason of a fall.

c) Inclusion criteria

1. Patients aged 75 and over.
2. Patients admitted for falls.
3. Clinically stable patient who may benefit from a gait assessment or falls risk assessment
4. Patients admitted to emergency from Monday 6am to Thursday 12pm.
5. Patients able to understand the protocol and not verbally object to participation.

d) Non-inclusion criteria

1. Patients with limb fractures.

2. Patients under guardianship or curatorship.

3. Patients already included in an interventional research protocol.

We have decided not to exclude patients for whom the etiology of the fall would immediately require hospitalization. Nevertheless, without specifying the reason for hospitalization, patients will be notified that their course of care is predefined by this etiology. This group of patients could be included in a secondary analysis of the impact of early rehabilitation.

Similarly, the discovery of another pathology in the ED or the decompensation of a known medical pathology are not reasons for non-inclusion. The care pathway will also be oriented towards hospitalization, which will be reported, but allowing the inclusion of these patients will enable us to gain an overall view of the physiotherapy activity in emergency departments for patients with falls.

e) Forecast duration

A 30-minute intervention time is planned, per patient, per day. All patients in the "Bilan MK" group will receive a BDK. Rehabilitation sessions will continue every weekday in hospital, until discharge from the emergency department. The number of rehabilitation sessions will therefore vary from patient to patient.

The inclusion phase will last 6 months, in order to be representative of the activity of MKs in the emergency departments of the CHU de Nantes. Patient follow-up will be based on a study of the patient's course of care, from admission to the emergency department up to 7 days after discharge (assessment of recurrence).

Data processing, formatting of results and submission of a publication in a leading peer-reviewed international journal (SIGAPS A or B, or Quartile 1 in rehabilitation, geriatrics or emergency medicine) will take 12 months.

Biological collection or retention of samples beyond the duration of the study is planned: yes ☐ no ☒

3. Absence of Research Risks and Constraints

The proposed research involves no risk to patients or caregivers other than those associated with current practice. As such, any adverse events or effects observed will be unrelated to the study.

4. Personal data protection

In compliance with the RGPD, we plan to collect the data below:

- Patient's age and sex
- Output orientation

- Length of stay
- Recurrence within 7 days of discharge
- Physician satisfaction (see Appendix 2)
- Medical discharge decision before/after MK assessment

We have decided not to exclude patients for whom the etiology of the fall would immediately require hospitalization. However, they will be notified that their care pathway is predefined by this etiology.

None of the clinical data from the MK assessment can be collected in order to position us outside the Jardé law.

The data collected are adequate, relevant and limited to what is necessary with regard to the research objectives: yes ☒ no ☐

5. Recruiting and informing people

a) Participant recruitment procedures (procedures, documents, cooling-off periods)

Patients aged 75 and over, hospitalized in the emergency department for a fall, will be offered the study. In the event of difficulty in obtaining non-opposition (patient unable to give non-opposition), the patient will not be included.

b) Information and non-opposition

An information note will be given to the patient in order to obtain his or her non-opposition.

c) Special populations

The research requires the inclusion of special populations: yes ☐ no ☒

If yes, please explain :

The conditions of article L. 1122-2 of the French Public Health Code are respected, in cases where the research involves unemancipated minors, protected adults or adults unable to express their consent: yes ☐ no ☐ not concerned ☐

The conditions of article L.1121-5 of the Public Health Code are met, in the event that the research involves pregnant or breastfeeding women: yes ☐ no ☐ not concerned ☐

6. Statistics and publication

a) Statistics

Statistical analyses will be carried out by a biostatistician from the Nantes University Hospital Biometrics Platform. Analyses will be performed using SAS 9.4® software.

Patient characteristics will be described at the time of study inclusion, globally and by randomization arm.

Randomization will be open-label, with a 1:1 ratio, and will be balanced by blocks of variable size. Randomization will be carried out using ENNOV Clinical software via the website: https://nantes-lrsy.hugo-online.fr/CSOnline/.

Connection is via a login, password and study number (NTLRXXX), issued by a data manager in the Nantes University Hospital Research Promotion Department.

The following information is required:

- Age (years)
- Compliance with inclusion and non-inclusion criteria (yes/no)
- No opposition to participation in the study (yes/no).

The randomization arm will be assigned automatically during randomization. An e-mail confirmation will be sent to the person who performed the randomization and to all the people concerned.
The randomization list will be drawn up by a statistician from the Nantes University Hospital Research Promotion Department. An explanatory guide to randomization will be available online under ENNOV Clinical.

Primary objective: patient orientation at emergency department discharge will be compared between the 2 groups using a Chi-2 test.

Secondary objectives: lengths of stay in the SAU and UHCD and of hospitalization will be compared between the 2 groups using Student's t-tests. The 7-day fall recurrence rate will be compared between the 2 groups using a Chi-2 test. Physicians' satisfaction with MK management in the emergency department will be described. In the "MK assessment" group, changes in referral decisions before and after MK assessment will be described.

The main analysis population will be the Intent-to-Treat population, corresponding to all randomized patients. The statistical significance threshold is set at 5%. Any missing data will be described and explained. There will be no imputation on missing data.

b) Publication

The study will be registered on an open-access website (Clinical trial) before the 1st patient is included in the study.

All information resulting from this study is considered confidential, at least until appropriate analysis and control by the study sponsor, coordinator and statistician have been completed.

Publications and scientific reports corresponding to this study will be produced under the responsibility of the study coordinator. The study coordinator will be the principal signatory of the communication and the editor of the documents, and will necessarily appear as the first or last author. The coordinating investigator draws up the list of authors. The study statistician will be listed as the third author.

LIST OF APPENDICES

- **APPENDIX 1:** Bibliographical references
- **APPENDIX 2:** Physician satisfaction questionnaire
- **APPENDIX 3:** Background notes

APPENDIX 1: BIBLIOGRAPHICAL REFERENCES

Boisguérin, B., & Valdelièvre, B. (2014). Urgences : la moitié des patients restent moins de deux heures, sauf ceux maintenus en observation *DREES - Etudes et résultats* (pp. 8).

Braun, F., Tazarourte, K., Leveneur, A., Tortiget, D., (2022) Mission Flash sur les urgences et soins non programmés. Retrived from https://solidarites-sante.gouv.fr/IMG/pdf/rapport_du_docteur_braun_-_mission_flash_on_emergencies_and_non-program_care.pdf

Cour des Comptes. (2019). Les urgences hospitalières: des services toujours trop sollicités (Vol. Tome 2, pp. 29).

Crane, J., & Delany, C. (2013). Physiotherapists in emergency departments: responsibilities, accountability and education. *Physiotherapy, 99*(2), 95-100. doi: 10.1016/j.physio.2012.05.003

Desmeules, F., Roy, J. S., MacDermid, J. C., Champagne, F., Hinse, O., & Woodhouse, L. J. (2012). Advanced practice physiotherapy in patients with musculoskeletal disorders: a systematic review. *BMC Musculoskelet Disord, 13*, 107. doi: 10.1186/1471-2474-13-107

DRESS. (2021). The medicine medicine. The establishments of establishments. https://drees.solidarites- sante.gouv.fr/sites/default/files/2021-07/Fiche%2025%20-%20La%20m%C3%A9decine%20d%E2%80%99urgence.pdf

Fruth, S. J., & Wiley, S. (2016). Physician Impressions of Physical Therapist Practice in the Emergency Department: Descriptive, Comparative Analysis Over Time. *Phys Ther, 96*(9), 1333-1341. doi: 10.2522/ptj.20150306

HAS. (2009). *Evaluation and management of elderly people who suffer repeated falls*. Retrieved from h t t p s : / / w w w . h a s - s a n t e . f r / j c m s / c _ 7 9 3 3 7 1 / f r / e v a l u a t i o n - e t - p r i s e - e n - c h a r g e - d e s - p e r s o n n e s - a g e e s - f a i s a n t - d e s - chutes-repetees.

JORF. (2016). *Loi de modernisation de notre système de santé n°0022 du 27 janvier 2016,*. Retrieved from https://www.legifrance.gouv.fr/codes/id/LEGIARTI000031930031/2016-01-28.

Lafrance, S., Demont, A., Thavorn, K., Fernandes, J., Santaguida, C., & Desmeules, F. (2021). Economic evaluation of advanced practice physiotherapy models of care: a systematic review with meta-analyses. *BMC Health Serv Res, 21*(1), 1214. doi: 10.1186/s12913-021-07221-6

Lefmann, S. A., & Sheppard, L. A. (2014). Perceptions of emergency department staff of the role of physiotherapists in the system: a qualitative investigation. *Physiotherapy, 100*(1), 86-91. doi: 10.1016/j.physio.2013.08.004

Matifat, E., Mequignon, M., Cunningham, C., Blake, C., Fennelly, O., & Desmeules, F. (2019). Benefits of Musculoskeletal Physical Therapy in Emergency Departments: A Systematic Review. *Phys Ther, 99*(9), 1150-1166. doi: 10.1093/ptj/pzz082

Manckoundia P, Ntari Soungui E, Tavernier-Vidal B, Mourey F. (2014). Psychomotor maladjustment syndrome. *Geriatr Psychol Neuropsychiatr Vieil,*12(1) :94-100 doi:10.1684/pnv.2014.0450

Mesnier, T., & Carli, P. (2019). Pour un pacte de refondation des urgences. In M. d. S. e. d. l. Santé (Ed.), (pp. 259).

Rall, G. (2016). Integration of masseur-physiotherapists in emergency departments in France: Preliminary study. *Techniques Hospitalières (Fédération Hospitalière de France), 760*, 59-64.

Sheppard, L. A., Anaf, S., & Gordon, J. (2010). Patient satisfaction with physiotherapy in the emergency department. *Int Emerg Nurs, 18*(4), 196-202. doi: 10.1016/j.ienj.2009.11.008

Stanhope, J., Grimmer-Somers, K., Milanese, S., Kumar, S., & Morris, J. (2012). Extended scope physiotherapy roles for orthopedic outpatients: an update systematic review of the literature. *J Multidiscip Healthc, 5*, 37-45. doi: 10.2147/JMDH.S28891

Taylor, N. F., Norman, E., Roddy, L., Tang, C., Pagram, A., & Hearn, K. (2011). Primary contact physiotherapy in emergency departments can reduce length of stay for patients with peripheral musculoskeletal injuries compared with secondary contact physiotherapy: a prospective non-randomised controlled trial. *Physiotherapy, 97*(2), 107-114. doi: 10.1016/j.physio.2010.08.011

APPENDIX 2: SASTIFACTION PHYSICIAN QUESTIONNAIRE

1) Do you consider the massage-kinesthesia (MK) assessment to be relevant to your care?

☐ Yes ☐ No

2) Did the MK assessment help you refine your discharge decision?

Not at all — Very much

0	1	2	3	4	5	6	7	8	9	10

3) On a scale of 0 to 10, how satisfied are you with MK care in emergency departments?

Dissatisfied — Very satisfied

0	1	2	3	4	5	6	7	8	9	10

4) Would you use masseur-physiotherapists more often after this study?

☐ Yes ☐ No

5) In your opinion, what is the added value of physiotherapy in emergency departments?

6) What are your expectations regarding physical therapy in emergency departments?
(Free response)

APPENDIX 3: INFORMATION NOTE

Information note for research participation
"Masseur-KInésithérapeute en service d'URGEnces: quel(s) impact(s) a l'intervention du MK sur le parcours de soins de la personne âgée chuteuse?"

Short title: KiUrge

Sponsor: CHU de Nantes
Study no.: RC23_0068

This document is given to the person participating in the research.

Dear Sir/Madam,

The Centre Hospitalier et Universitaire de Nantes wishes to carry out research for which it is the promoter (i.e. it is responsible for and organizes the research).

The aim of this study (KiUrge) is to assess the impact of the intervention of a physiotherapist in emergency departments, on the care pathway of elderly people suffering from falls. This study is being carried out over 6 months. Any person aged 75 or over, admitted to the emergency department for a fall, will be offered this study. A group of patients will be randomly assigned to you. One group will not be assessed by a physiotherapist, the other will. Emergency discharge referrals (return home, hospitalization, etc.) will be counted.

This search is based on data collected during the course of your care (age, gender, length of hospital stay) and information relating to the activities of healthcare professionals who have worked on your behalf (using procedure coding/traceability software).

Data from this study will be kept for 15 years after the end of the study.

This research is carried out in collaboration with the Motricité, Interactions, Performance laboratory (UR 4334) at Nantes University (Guillaume Le Sant, MK, PhD).

Your participation in the research, should you give your consent, can only be confirmed on condition that you meet all the inclusion criteria for participation in this research.

If the number of people planned for the study has been reached, you may not be included in the study despite having signed a consent form. In this case, you will not be followed up as part of the study, and any data concerning you will be destroyed. This will in no way affect the quality of your medical care.

This research poses no risk to your health. The results will be used for research purposes and will not provide any additional information about your health. The results will be used for research purposes and will not provide any additional information about your health, but will help to develop knowledge in the field of physiotherapy and its role in optimizing patient care.

DATA PROCESSING

The legal basis for processing your personal data is the public interest served by this research (Article 6.1.e of Regulation (EU) 2016/679 of the European Parliament and of the Council of 27 April 2016 on the protection of individuals with regard to the processing of personal data and on the free movement of such data (RGPD)). This is one of the exceptions provided for in Article 9 of the same text, allowing the processing of health data.

Processing complies with reference methodology MR004 published by the CNIL.

As part of this research, your personal data will be processed electronically: this will enable us to analyze the results of the research and fulfill the research objective.

For this purpose, your personal data will be collected in a computer file to which the Research Sponsor (Nantes University Hospital) will have access.

To ensure confidentiality, your data will be identified by a code number and your initials.

Persons involved in research as part of their duties will have access to your coded data.

The only people who will have access to your directly identifiable data are the people in charge of your care as part of the research and the people responsible for quality control and assurance of the research.

Your data may be transferred nationally or internationally (e.g. to persons or companies acting on behalf of the CHU, to authorized health authorities) for this study, or for other subsequent research, exclusively for scientific purposes, under conditions ensuring confidentiality, via secure platforms or servers. Within Europe, the protection of your data is guaranteed (EU Regulation 2016/679). Outside Europe, your data may be transferred to countries that do not have the same level of data protection requirements. In this case, the Promoter will take all necessary measures to protect the data collected and must undertake to ensure a level of security equivalent to that covered by French and European laws for data sent abroad. If you wish, you can obtain a copy of all the measures taken to ensure the protection of your data from the Data Protection Officer (DPO), see at the end of the document, paragraph "Your contacts".

Your pseudonymized data (identified by a code number and your initials) may be used in publications or communications; in this case, your anonymity will be preserved.

In accordance with the provisions of the French Data Protection Act (amended Act of January 6, 1978), and Regulation (EU) 2016/679 of the European Parliament and of the Council of April 27, 2016 on the protection of individuals with regard to the processing of personal data and on the free movement of such data (RGPD), you have the right to access, rectify, limit and object to the processing of your personal data. If you decide to withdraw your consent to participate in the research, the data obtained before your consent was withdrawn will be used in accordance with article L.1122-1-1 of the CSP. Data collected after you have withdrawn your consent will not be used for this research and will remain strictly for healthcare purposes. You may also lodge a complaint with a supervisory authority (CNIL for France: https://www.cnil.fr/fr/webform/adresser-une-plainte/).

This data may be used for further research, exclusively for scientific purposes. You may withdraw your consent to this further use or exercise your right to object at any time.

You can also access all your medical data directly or through a doctor of your choice, in accordance with article L 1111-7 of the French Public Health Code.

Your data will be kept for the duration of the research. Once the research has been completed, the data will be archived for 15 years and then destroyed.

At your request, the investigator can inform you of the overall results of this research.

To find out more, or to exercise your rights concerning your data, see "Your contacts" at the end of this document.

This search complies with :<br><br>- the French Data Protection Act of January 6, 1978, as amended, and Act no. 2018-493 of June 20, 2018 on the protection of personal data<br>- to Regulation (EU) 2016/679 of the European Parliament and of the Council of 27 April 2016 on the protection of individuals with regard to the processing of personal data and on the free movement of such data (RGPD)This project and this document were presented to the Nantes Group for Ethics in Health GNEDS.

Your participation in this research is voluntary. You may refuse to take part in this research, and you may withdraw from it at any time, without prejudice. This will have no effect on the quality of care you receive; you simply need to inform the investigator.

YOUR CONTACTS :

For any question concerning the study, withdrawal of consent, or to exercise your rights concerning your data (access, rectification, etc...), your preferred contact is :

**The coordinating investigator :**<br>BLANDIN Marie<br>✉ marie.blandin@chu-nantes.fr<br>☎ 02.40.08.33.33

For general questions about the processing of your data :

**The research sponsor, data controller :**<br>CHU de Nantes, direction de la recherche et de l'innovation<br>5 allée de l'Ile Gloriette, 44093 NANTES Cedex 1<br>**The Data Protection Officer (DPO) :**<br>*vosdonneespersonnelles@chu-nantes.fr*

**Please keep this information leaflet**

Titre de la recherche : masseur-KInésithérapeute en service d'URGEnces : quel(s) impact(s) a l'intervention du MK sur le parcours de soins de la personne âgée chuteuse ?

Acronyme de la recherche : KiUrge

Réf. : RC23_0068

Promoteur:

CHU de Nantes
Direction de la Recherche et de l'Innovation
5, allée de l'île Gloriette
44 093 Nantes cedex 01 (FRANCE)

Bp-prom-regl@chu-nantes.fr
Tel : 02 53 48 28 35
Fax : 02 53 48 28 36

Porteur du projet / Investigateur coordonnateur
Marie BLANDIN
Masseur-kinésithérapeute D.E., CHU de Nantes – Hôtel Dieu
marie.blandin@chu-nantes.fr

Investigateur associé
Marie GALLET (Masseur-kinésithérapeute D.E., CHU de Nantes – Hôtel Dieu)

Collaborateurs
Guillaume LE SANT (PhD PT, Université de Nantes)
Thomas RULLEAU (PT PhD, Coordonnateur paramédical de la recherche, CHU Nantes)
Christelle VOLTEAU (Méthodologiste et biostatisticienne, CHU Nantes)

- La recherche a pour objet le développement des connaissances biologiques ou médicales :

  oui ☐ non ☒

- La recherche est effectuée sous la direction et sous la surveillance d'un médecin ou d’une personne qualifiée justifiant d'une expérience appropriée: oui ☒ non ☐

Index des abréviations

BDK : Bilan-diagnostic kinésithérapique

MAG : Médecine Aigue Gériatrique

MK : Masseur-Kinésithérapeute

MPG : Médecine Polyvalente Gériatrique

MPU : Médecine Polyvalente d'Urgence

RAD : Retour à Domicile

SAU : Service d'Accueil des Urgences

UHCD : Unité d'Hospitalisation de Courte Durée

1. Intérêt et pertinence de la recherche

a) Contexte et justification

La sur-sollicitation des services d'urgences est une problématique majeure du système de santé français. Ces services concentrent les difficultés de ce dernier à répondre aux besoins de santé des populations. En 2019, plus de 22 millions de passages ont été recensés dans les 697 structures d'urgence (publiques et privées), un chiffre en constante progression : +15% par rapport à 2012, tandis « que » 10 millions de passages étaient observés vingt ans plus tôt en 1996 (Mesnier & Carli, 2019) (DRESS, 2021). Cette croissance crée une tension induite par cette sur-sollicitation, ce qui augmente le risque de dégradation de la qualité des soins prodigués aux patients et sur l'activité des soignants dans les services d'urgences. Afin de trouver une solution à cette problématique, le Pacte de Refondation des urgences (Rapport « Carli-Mesnier ») initié en 2019 ou la récente mission Flash sur les urgences et soins non programmés (Rapport « Braun ») en 2022 proposent plusieurs mesures en amont, en aval et au sein en services d'urgences. En particulier, le Rapport « Carli-Mesnier » propose de « Renforcer et reconnaître les compétences des professionnels des urgences » (Mesure 8).

Les masseurs-kinésithérapeutes (MK) interviennent dans certains pays anglo-saxons pour prendre en soins les patients admis en services d'urgence pour des problématiques de traumatologie bénigne depuis les années 1970. Les motifs orthopédiques (entorses, fractures…) ou rhumatologiques (douleurs persistantes type lombalgiques, arthrosiques…) sont les situations principales pour lesquels les MK sont sollicités (Stanhope et al., 2012). Les MK peuvent y tenir 2 rôles distincts : un rôle d'évaluation et/ou de traitement suite à une référence du médecin ou un rôle en première ligne de soins qui comprend l'accès direct du patient, l'élaboration du plan de traitement et le congé du patient (Desmeules et al., 2012 ; Matifat et al., 2019). Les résultats de ces interventions rapportent dans la littérature: une amélioration des coûts directs et indirects liés à la situation de santé (e.g. durée et services d'hospitalisation, consommation hors-hôpital), une sécurité des soins maintenue (lorsque le MK intervient en accès direct), et un degré de satisfaction des équipes/des patients important (Lafrance, S et al.,2021; Matifat, E et al.,2019; Fruth, SJ & Wiley, S.,2016; Morris, J et al.,2015; Taylor, N et al.,2011;Stanhope, J et al.,2012). Nous pouvons nous interroger sur d'autres domaines où la kinésithérapie pourrait être un recours intéressant. En complément de ces études sur la kinésithérapie en orthopédie et rhumatologie, Lefmann et Sheppard (2014) rapportent que la kinésithérapie pourrait avoir un intérêt dans d'autres domaines d'activités, en services d'urgence : en particulier en gérontologie (e.g. pour des problématiques liées aux chutes, à l'équilibre), ou en neurologie (e.g. pour des troubles de la déglutition…) (Lefmann & Sheppard, 2014).

Les prescriptions médicales réalisées dans les services d'urgences du CHU de Nantes vont dans ce sens puisqu'il est régulièrement prescrit aux MK des actes en lien avec la gérontologie, la déglutition, les vertiges. Cette perspective d'utiliser les compétences du MK auprès des populations âgées en services d'urgences apparaît comme une solution à explorer pour gérer les problématiques précédemment présentées. Cette réflexion est d'autant plus importante à considérer en tenant compte de la population accueillie aux urgences du CHU de Nantes. En effet, un relevé réalisé en mai 2022 sur les urgences du CHU de Nantes montre que :

- seulement 20 % des patients sont venus aux urgences pour des motifs en lien avec de la traumatologie bénigne (population de patients correspondant aux attentes identifiés dans la littérature anglo-saxonne).
- au contraire 42% sont des patients âgés hospitalisés pour motif de chute, soit en moyenne 13 patients par jour pour le service.

Les patients âgés de 75 ans et plus constituent une patientèle particulière des services d'urgences à plusieurs titres. La fragilité, la dépendance et les polypathologies, plus fréquentes parmi ces patients, justifient une prise en soins plus complexe que pour les autres patients et entraînent des durées de passage plus longues (Boisguérin & Mauro, 2017). L'admission par les urgences est un facteur de risque important de dégradation pour les personnes âgées : 23 % des 75 ans et plus passent plus de 8h aux urgences et sont beaucoup plus sujettes à la survenue d'événements indésirables lors de leur passage (Mesnier & Carli, 2019). Pour ces patients, il est actuellement demandé aux MK du service de réaliser des évaluations de l'autonomie et du risque de chute. Les prescriptions visent à élaborer un parcours de soin optimal pour ces patients. Ces évaluations permettent de compléter le diagnostic médical initial et visent à donner de nouveaux arguments aux médecins pour établir leurs décisions de sortie. À l'issue de l'admission au Service d'Accueil des Urgences (SAU), trois décisions médicales de sortie sont possibles pour ces patients : un retour à domicile (RAD), une hospitalisation dans l'Unité d'Hospitalisation de Courte Durée (UHCD) qui ne doit pas dépasser 48h ou une hospitalisation plus longue dans un des services de la filière gériatrique (Médecine aigue gériatrique (MAG), Médecine polyvalente d'urgence (MPU), Médecine polyvalente gériatrique (MPG), Soins de Suite et Réadaptation….) (cf. Figure 1).

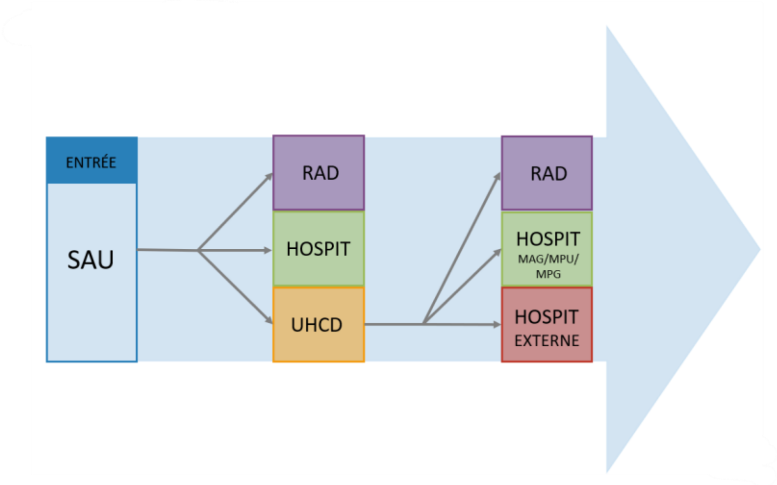

Figure 1- Parcours de soin possibles pour les personnes âgées chuteuses admises au SAU et à l'UHCD (SAU : Services d'Accueil des Urgences, RAD : Retour A Domicile, UHCD : Unité d'Hospitalisation de Courte Durée)

Le bilan-diagnostic kinésithérapique (BDK) initial peut jouer un rôle dans l'orientation du parcours de soins.

À partir de ces données issues de recherche aux urgences sur la traumatologie, adaptées au contexte nantais où le pourcentage de passage aux urgences des patients de 75 ans et plus pour chute est élevé, nous questionnons l'impact de l'action du MK (BDK +/- séance) sur le parcours du patient.

Le BDK initial peut s'effectuer à deux instants :

- Au SAU
- A l'UHCD, lorsque les patients y sont transférés rapidement après leur admission au SAU.

Nous souhaitons évaluer l'impact du BDK, réalisé au SAU ou à l'UHCD, sur la décision médicale de sortie.

b) Objectif principal et critère de jugement principal

L'objectif principal de cette étude est de comparer l'orientation des patients à la sortie des services d'urgence entre les patients bénéficiant d'un bilan-diagnostic MK et les patients sans bilan MK.

Le critère de jugement principal est l'orientation du patient à la sortie du SAU et de l'UHCD, selon les parcours définis en aval (RAD/UHCD/hospitalisation pour les inclusions réalisées au SAU ou RAD/hospitalisation pour les inclusions réalisées à l'UHCD).

c) Objectifs secondaires et critères de jugement secondaires

<u>Objectifs secondaires :</u>

1. Comparer la durée de séjour au sein des structures d'urgence entre les deux groupes de patients (bénéficiant de MK vs ne bénéficiant pas de MK).
2. Comparer la durée de séjour globale à l'hôpital entre les deux groupes de patients.
3. Comparer le taux de récidive de chute entre les 2 groupes.
4. Evaluer la satisfaction des médecins intervenant au SAU et à l'UHCD par rapport à l'apport de la masso-kinésithérapie dans la prise en soins des patients.
5. Dans le groupe de patients avec évaluation MK, comparer la décision médicale d'orientation à la sortie du SAU et de l'UHCD, avant et après bilan MK.

<u>Critères de jugement secondaires :</u>

1. Durée de séjour au sein du SAU et UHCD : calcul de la durée en heures par un relevé des dates et des heures d'admission et de sortie du service.
2. Durée totale d'hospitalisation pour chaque séjour, tous services confondus : calcul de la durée en heures par un relevé des dates et des heures d'admission aux SAU et de sortie définitive du CHU.
3. Récidive d'admission dans un service d'urgences, pour le même motif, de la personne âgée dans les 7 jours suivant son retour à domicile. Un appel téléphonique sera passé au patient ou à sa famille à 7 jours de sa sortie à domicile. Les questions posées seront : « Avez-vous rechuté depuis votre retour à domicile ? Si oui, combien de fois ? Avez-vous été admis aux urgences pour ce motif ? »
4. Satisfaction des médecins du SAU. Pour évaluer cette satisfaction, un questionnaire standardisé sera transmis à l'ensemble des médecins ayant participé à prendre en soins les patients de l'étude (Annexe 2).
5. Orientation à la sortie du SAU et de l'UHCD avant et après évaluation par le MK.

2. Organisation de l'étude

a) Schéma de l'étude

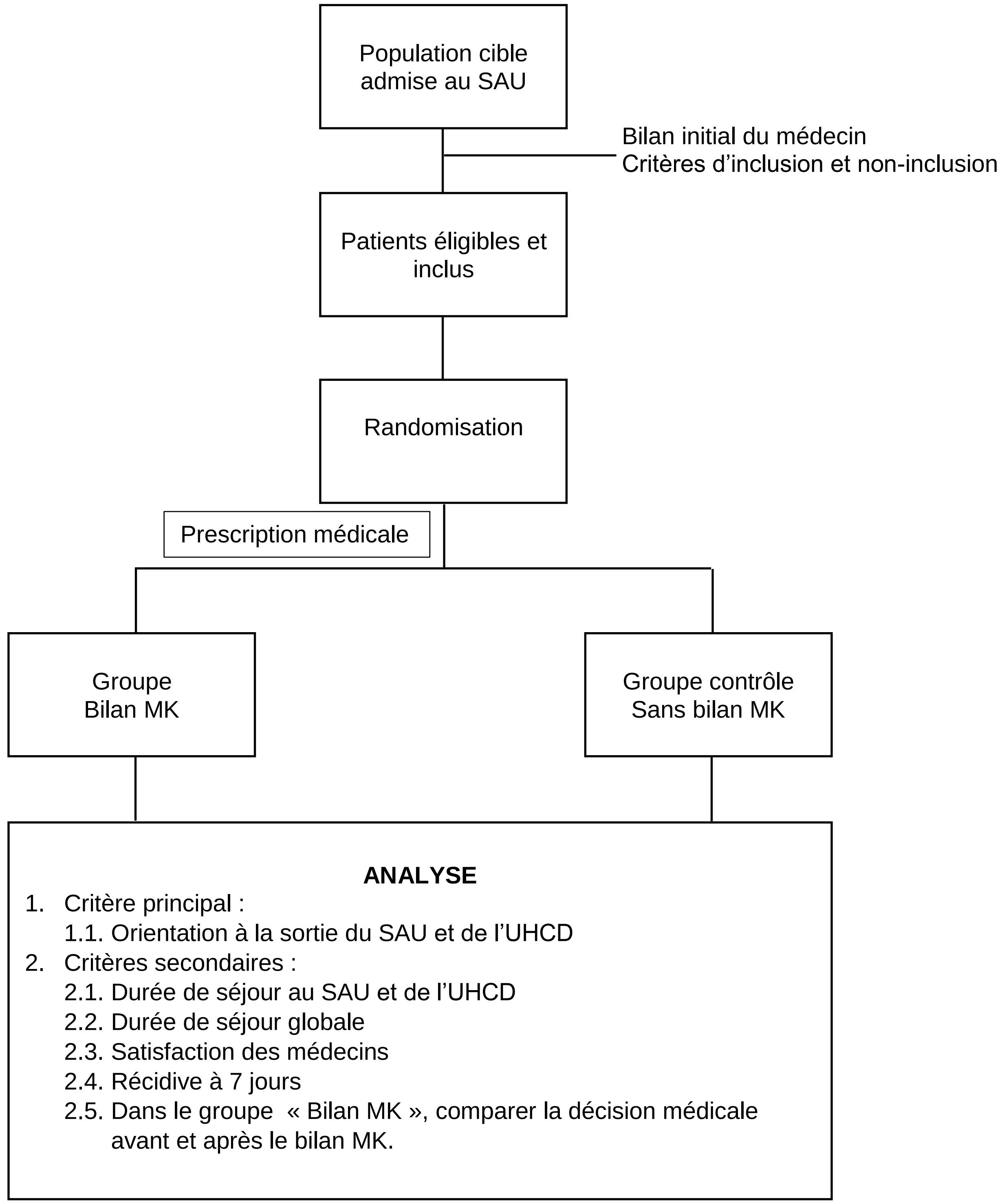

Description du Bilan MK :

Selon les recommandations de l'HAS de 2009 (dernières recommandations en ligne), les actes et techniques suivants seront appliqués durant le bilan :

Acte/Procédure	Technique / conditions	Fréquence	Durée
**Bilan MK**	Test de Tinetti	1 fois	10 min
	Time Up and Go test	1 fois	10 min
	Test unipodal	1 fois	2 min
	Romberg	1 fois	2 min
	Stop walking when talking test	1 fois	1 min
	Bilan articulaire MI	1 fois	5 min
**Séance de rééducation**	En fonction du bilan	Chaque jour d'hospitalisation	30 min

b) Méthode

Les caractéristiques principales de la recherche sont les suivantes :

- Etude pilote monocentrique hors loi Jardé.

  Le positionnement hors loi Jardé est motivé par le fait que la recherche n'a pas pour objectif le développement des connaissances biologiques ou médicales et de ce fait, l'absence de récolte de données de santé dans les critères d'évaluation des patients.

- Etude prospective, randomisée, contrôlée (ratio 1 : 1) : patients avec bilan MK systématique après la consultation médicale versus patients sans bilan MK.

Après cette étude pilote à court terme monocentrique qui vise à s'assurer la faisabilité du protocole et de récolter des données, nous souhaitons permettre une étude de plus grande envergure questionnant l'intérêt de la kinésithérapie aux urgences au niveau national et à moyen/ long terme sur le parcours patient et son impact médico-économique.

Déroulé de l'étude

Il s'agit d'une étude pilote dont le but est d'évaluer les effets d'un bilan-diagnostic MK après la consultation médicale à l'arrivée aux urgences. Le critère principal de l'étude est l'orientation du patient à la sortie de SAU et UHCD.

Une alerte informatique sera mise en place sur FirstNet, pour que le TEC puisse effectuer un screening des patients éligibles. Firstnet est un logiciel qui permet d'avoir une liste des patients admis au SAU, en fonction des secteurs. Pour chacun d'eux, une équipe médicale et paramédicale y est renseignée.

L'inclusion pourra avoir lieu au SAU ou éventuellement à l'UHCD lorsque les patients y sont transférés avant d'avoir pu être inclus et évalués au SAU. Par exemple, un patient admis au SAU à 18h et transféré dans la nuit à l'UHCD pourra être inclus et randomisé uniquement à l'UHCD.

Pour tout patient éligible, il faudra s'assurer auprès du référent médical que le patient est stable cliniquement et apte à bénéficier d'un bilan de marche ou de risque de chute.

La randomisation sera effectuée par le TEC. Le médecin effectuera une prescription pour les patients inclus dans le groupe « Bilan MK ».

Pour un patient non inclus, le médecin qui souhaite un BDK pourra le prescrire et celui-ci sera réalisé selon les possibilités actuelles (hors étude).

Afin de diminuer les biais en lien avec l'organisation hebdomadaire des services, les inclusions se feront auprès de patients entrant aux urgences du lundi matin au jeudi midi. En effet, les durées de séjour peuvent être influencées par les admissions pour les autres jours de la semaine, en particulier le week-end (moins de personnel, moins de transferts inter-services, …). Aussi, le week-end, seules les kinésithérapies respiratoires sont effectuées, le MK de garde ne peut donc pas réaliser les évaluations en lien avec le projet.

c) Effectif (justification, hypothèses, échantillonnage, en fonction de la nature de l'étude)

Il n'y a pas de données dans la littérature qui permettent de justifier le nombre de patients à inclure dans l'étude. L'objectif est donc de recruter de manière la plus exhaustive possible l'ensemble des patients répondant aux critères d'inclusion et de non inclusion de l'étude sur une période d'inclusion suffisamment longue (6 mois) permettant d'être représentatif de la population des personnes âgées chuteuses admises aux urgences du CHU de Nantes. L'analyse préliminaire des passages aux urgences du CHU de Nantes (en mai 2022) permet d'envisager, quotidiennement environ 13 patients âgés chuteurs.

Sur 6 mois d'inclusion, la capacité d'inclusion théorique sur cette population serait d'environ 1092 patients (13 patients x 3.5 jours x 24 semaines = 1092 patients).

Actuellement, 20 % de temps de kinésithérapie sont alloués aux services d'urgence, soit environ 3 patients vus par jour.

Afin de poursuivre les prises en charge déjà existantes (kinésithérapies respiratoires, bilans de déglutition…), tout en permettant le bon déroulement de cette étude, il est prévu de financer 30% de temps MK supplémentaires. Ceci pourra nous permettre d'inclure 4 patients par jour, soit **336 patients** (4 patients x 3.5 jours x 24 semaines = 336 patients).

b) Population de l'étude, sujets étudiés

La population concernée par cette étude est celle de 75 ans et plus admise au SAU pour un motif de chute.

c) Critères d'inclusion

1. Patients de 75 ans et plus.
2. Patients admis pour chute.
3. Patient stable cliniquement pouvant bénéficier d'un bilan de marche ou de risque de chute
4. Patients admis aux urgences du lundi matin 6h au jeudi 12h.
5. Patients aptes à comprendre le protocole et ne pas s'opposer oralement à sa participation.

d) Critères de non-inclusion

1. Patients présentant des fractures de membres.

2. Patients sous tutelle ou sous curatelle.

3. Patients déjà inclus dans un protocole de recherche interventionnelle.

Nous décidons de ne pas exclure secondairement les patients pour lesquels l'étiologie de la chute nécessiterait d'emblée une hospitalisation. Néanmoins, sans préciser le motif d'hospitalisation, il sera notifié que son parcours de soin est prédéfini par cette étiologie. Ce groupe de patients pourra faire partie d'une analyse secondaire, sur l'impact de la rééducation précoce.

De même, la découverte d'une autre pathologie au SAU ou la décompensation d'une pathologie médicale connue ne sont pas des motifs de non inclusion. Le parcours de soin sera également orienté vers une hospitalisation, ce qui sera notifié, mais permettre l'inclusion de ces patients nous permettra d'avoir une vision globale sur l'activité de kinésithérapie aux urgences chez les patients chuteurs.

e) Durée prévisionnelle

Il est prévu un temps d'intervention de 30 minutes, par patient et par jour. Tous les patients du groupe « Bilan MK » bénéficieront d'un BDK. Les séances de rééducation se poursuivront chaque jour d'hospitalisation de semaine, jusqu'à la sortie des services d'urgences. Le nombre de séances de rééducation sera donc susceptible de varier d'un patient à un autre.

La phase d'inclusion se fera sur 6 mois, pour être représentative de l'activité des MK au sein des services d'urgences du CHU de Nantes. Le suivi du patient se fera à partir de l'étude de son parcours de soin, de son entrée au SAU jusqu'à 7 jours après sa sortie d'hospitalisation (évaluation de la récidive).

La phase de traitement des données, de mise en forme des résultats et de soumission d'une publication dans une revue internationale à comité de lecture de premier plan (SIGAPS A ou B, ou Quartile 1 en rééducation, gériatrie ou médecine d'urgences) se fera sur une durée de 12 mois.

Une collection biologique ou la conservation d'échantillons au-delà de la durée de l'étude est prévue : oui ☐ non ☒

3. Absence de Risques et de contraintes de la recherche

La recherche proposée ne comporte aucun risque pour les patients ou les soignants en dehors de ceux en lien avec la pratique courante. En ce sens, les évènements ou effets indésirables éventuellement observés seront sans lien avec l'étude.

4. Protection des données personnelles

En conformité avec le RGPD, nous prévoyons de récolter les données ci-dessous :

- Age et sexe du patient
- Orientation de sortie

- Durée de séjour
- Récidive à 7 jours de la sortie
- Satisfaction médecins (cf. Annexe 2)
- Décision médicale de sortie avant/après bilan MK

Nous décidons de ne pas exclure secondairement les patients pour lesquels l'étiologie de la chute nécessiterait d'emblée une hospitalisation. Néanmoins, il sera notifié que son parcours de soin est prédéfini par cette étiologie.

Aucune des données cliniques issues du bilan MK ne pourra être récoltée afin de nous positionner hors loi Jardé.

Les données collectées sont adéquates, pertinentes et limitées à ce qui est nécessaire au regard des objectifs de la recherche : oui ☒ non ☐

5. Recrutement et information des personnes

a) Modalités de recrutement des participants (procédures, documents, délais de réflexion)

Les patients âgés de 75 ans et plus, hospitalisés aux urgences pour chute se verront proposer l'étude. En cas de difficulté à recueillir la non-opposition (patient hors d'état de donner sa non-opposition), le patient ne sera pas inclus.

b) Information et non-opposition

Une note d'information sera remise au patient afin de recueillir sa non-opposition.

c) Populations particulières

La recherche nécessite l'inclusion de populations particulières : oui ☐ non ☒

Si oui, justifier :

Les conditions de l'article L. 1122-2 du Code de la santé publique sont respectées, dans le cas où la recherche porte sur des mineurs non émancipés, des majeurs protégés ou des majeurs hors d'état d'exprimer leur consentement : oui ☐ non ☐ non concerné ☐

Les conditions de l'article L.1121-5 du Code de la santé publique sont respectées, dans le cas où la recherche porte sur des femmes enceintes ou allaitantes : oui ☐ non ☐ non concerné ☐

6. Statistiques et publication

a) Statistiques

Les analyses statistiques seront réalisées par un biostatisticien de la Plateforme de Biométrie du CHU de Nantes. Les analyses seront réalisées sous le logiciel SAS 9.4®.

Les caractéristiques des patients seront décrites à leur inclusion dans l'étude globalement et par bras de randomisation.

La randomisation sera effectuée en ouvert, selon un ratio 1 : 1 et sera équilibrée par blocs de tailles variables. La randomisation sera effectuée via le logiciel ENNOV Clinical par la connexion au site internet : **https://nantes-lrsy.hugo-online.fr/CSOnline/**.

La connexion s'effectuera grâce à un login, un mot de passe et un numéro d'étude (NTLRXXX), délivrés par un data-manager du Département Promotion de la Recherche du CHU de Nantes.

Les informations suivantes devront être renseignées :

- Age (années)
- Respect des critères d'inclusion et de non inclusion (oui/non)
- Non opposition à la participation à l'étude (oui/non).

Le bras de randomisation sera attribué automatiquement lors de la randomisation. Une confirmation par mail sera envoyée à la personne ayant effectuée la randomisation ainsi qu'à toutes les personnes concernées.
La liste de randomisation sera réalisée par un statisticien du département Promotion de la Recherche du CHU de Nantes. Un guide explicatif de la randomisation sera disponible en ligne sous ENNOV Clinical.

Objectif principal : l'orientation des patients à la sortie des urgences sera comparée entre les 2 groupes par un test du Chi-2.

Objectifs secondaires : les durées de séjours aux SAU et UHCD et d'hospitalisation seront comparées entre les 2 groupes via des tests de Student. Le taux de récidive de chute à 7 jours sera comparé entre les 2 groupes avec un test du Chi-2. La satisfaction des médecins pour la prise en charge MK aux urgences sera décrite. Dans le groupe « Bilan MK », les modifications de décision d'orientation avant-après bilan MK seront décrites.

La population d'analyse principale sera la population en Intention de Traiter correspondant à l'ensemble des patients randomisés. Le seuil de significativité statistique est fixé à 5%. Les éventuelles données manquantes seront décrites et explicitées. Il n'y aura pas d'imputation effectuée sur les données manquantes.

b) Publication

L'étude sera enregistrée sur un site web en libre accès (Clinical trial) avant l'inclusion du 1er patient dans cette étude.

Toutes les informations résultant de cette étude sont considérées comme confidentielles, au moins jusqu'à ce que l'analyse appropriée et le contrôle par le promoteur, le coordonnateur et le statisticien de l'étude soient achevés.

Les publications et rapports scientifiques correspondant à cette étude seront réalisés sous la responsabilité du coordonnateur de l'étude. Le coordonnateur de l'étude sera le signataire principal de la communication et le rédacteur des documents, il figurera nécessairement au rang du premier ou dernier auteur. L'investigateur coordonnateur établit la liste des auteurs. Le statisticien de l'étude sera cité en troisième auteur.

LISTE DES ANNEXES

- **ANNEXE 1 :** Références bibliographiques
- **ANNEXE 2 :** Questionnaire satisfaction des médecins
- **ANNEXE 3 :** Notes d'information

ANNEXE 1 : REFERENCES BIBLIOGRAPHIQUES

Boisguérin, B., & Valdelièvre, B. (2014). Urgences : la moitié des patients restent moins de deux heures, hormis ceux maintenus en observation *DREES - Etudes et résultats* (pp. 8).

Braun, F., Tazarourte, K., Leveneur, A., Tortiget, D., (2022) Mission Flash sur les urgences et soins non programmés. Retrived from https://solidarites-sante.gouv.fr/IMG/pdf/rapport_du_docteur_braun_-_mission_flash_sur_les_urgences_et_soins_non_programmes.pdf

Cour des Comptes. (2019). Les urgences hospitalières : des services toujours trop sollicités (Vol. Tome 2, pp. 29).

Crane, J., & Delany, C. (2013). Physiotherapists in emergency departments: responsibilities, accountability and education. *Physiotherapy, 99*(2), 95-100. doi: 10.1016/j.physio.2012.05.003

Desmeules, F., Roy, J. S., MacDermid, J. C., Champagne, F., Hinse, O., & Woodhouse, L. J. (2012). Advanced practice physiotherapy in patients with musculoskeletal disorders: a systematic review. *BMC Musculoskelet Disord, 13*, 107. doi: 10.1186/1471-2474-13-107

DRESS. (2021). La médecine d'urgence. Les établissements de santé. https://drees.solidarites-sante.gouv.fr/sites/default/files/2021-07/Fiche%2025%20-%20La%20m%C3%A9decine%20d%E2%80%99urgence.pdf

Fruth, S. J., & Wiley, S. (2016). Physician Impressions of Physical Therapist Practice in the Emergency Department: Descriptive, Comparative Analysis Over Time. *Phys Ther, 96*(9), 1333-1341. doi: 10.2522/ptj.20150306

HAS. (2009). *Evaluation et prise en charge des personnes âgées faisant des chutes répétées*,. Retrieved from https://www.has-sante.fr/jcms/c_793371/fr/evaluation-et-prise-en-charge-des-personnes-agees-faisant-des-chutes-repetees.

JORF. (2016). *Loi de modernisation de notre système de santé n°0022 du 27 janvier 2016,*. Retrieved from https://www.legifrance.gouv.fr/codes/id/LEGIARTI000031930031/2016-01-28.

Lafrance, S., Demont, A., Thavorn, K., Fernandes, J., Santaguida, C., & Desmeules, F. (2021). Economic evaluation of advanced practice physiotherapy models of care: a systematic review with meta-analyses. *BMC Health Serv Res, 21*(1), 1214. doi: 10.1186/s12913-021-07221-6

Lefmann, S. A., & Sheppard, L. A. (2014). Perceptions of emergency department staff of the role of physiotherapists in the system: a qualitative investigation. *Physiotherapy, 100*(1), 86-91. doi: 10.1016/j.physio.2013.08.004

Matifat, E., Mequignon, M., Cunningham, C., Blake, C., Fennelly, O., & Desmeules, F. (2019). Benefits of Musculoskeletal Physical Therapy in Emergency Departments: A Systematic Review. *Phys Ther, 99*(9), 1150-1166. doi: 10.1093/ptj/pzz082

Manckoundia P, Ntari Soungui E, Tavernier-Vidal B, Mourey F. (2014). Syndrome de désadaptation psychomotrice. *Geriatr Psychol Neuropsychiatr Vieil,*12(1) :94-100 doi:10.1684/pnv.2014.0450

Mesnier, T., & Carli, P. (2019). Pour un pacte de refondation des urgences. In M. d. S. e. d. l. Santé (Ed.), (pp. 259).

Rall, G. (2016). Intégration des masseurs kinésithérapeutes dans les services d'urgence en France : Étude préliminaire. *Techniques Hospitalières (Fédération Hospitalière de France), 760*, 59-64.

Sheppard, L. A., Anaf, S., & Gordon, J. (2010). Patient satisfaction with physiotherapy in the emergency department. *Int Emerg Nurs, 18*(4), 196-202. doi: 10.1016/j.ienj.2009.11.008

Stanhope, J., Grimmer-Somers, K., Milanese, S., Kumar, S., & Morris, J. (2012). Extended scope physiotherapy roles for orthopedic outpatients: an update systematic review of the literature. *J Multidiscip Healthc, 5*, 37-45. doi: 10.2147/JMDH.S28891

Taylor, N. F., Norman, E., Roddy, L., Tang, C., Pagram, A., & Hearn, K. (2011). Primary contact physiotherapy in emergency departments can reduce length of stay for patients with peripheral musculoskeletal injuries compared with secondary contact physiotherapy: a prospective non-randomised controlled trial. *Physiotherapy, 97*(2), 107-114. doi: 10.1016/j.physio.2010.08.011

ANNEXE 2 : QUESTIONNAIRE SASTIFACTION MEDECIN

1) Le bilan masso-kinésithérapique (MK) vous a-t-il paru pertinent dans la prise en charge ?

☐ Oui ☐ Non

2) Le bilan MK vous a-t-il permis d'affiner votre décision de sortie ?

Pas du tout — Beaucoup

0	1	2	3	4	5	6	7	8	9	10

3) Sur une échelle de 0 à 10, quel est votre niveau de satisfaction concernant la prise en charge MK au sein des services d'urgence ?

Insatisfait — Très satisfait

0	1	2	3	4	5	6	7	8	9	10

4) Feriez-vous plus appel aux masseurs-kinésithérapeutes après cette étude ?

☐ Oui ☐ Non

5) Selon vous, quelle est la plus-value de la kinésithérapie au sein des services d'urgences ? (Réponse libre)

6) Quelles sont vos attentes concernant la kinésithérapie au sein des services d'urgences ? (Réponse libre)

ANNEXE 3 : NOTE D'INFORMATION

Note d'information pour la participation à la recherche
« Masseur-KInésithérapeute en service d'URGEnces : quel(s) impact(s) a l'intervention du MK sur le parcours de soins de la personne âgée chuteuse ? »

Titre abrégé : « KiUrge»

Promoteur : CHU de Nantes
N° de l'étude : RC23_0068

Ce document est remis à la personne participant à la recherche

Madame, Monsieur,

Le Centre Hospitalier et Universitaire de Nantes souhaite mener une recherche dont il est le promoteur (c'est à dire qu'il en est responsable et qu'il l'organise).

L'objectif de cette étude (KiUrge) est d'appréhender l'impact de l'intervention d'un kinésithérapeute au sein des services d'urgence, sur le parcours de soin de la personne âgée chuteuse. Cette étude est réalisée sur 6 mois. Toute personne de 75 ans ou plus, admise au service d'accueil des urgences pour chute, se verra proposer cette étude. Un groupe de patients vous sera attribué au hasard. L'un des groupes ne sera pas bilanté par un kinésithérapeute, l'autre le sera. Les orientations de sortie des urgences (retour à domicile, hospitalisation…) seront comptabilisées.

Cette recherche est réalisée à partir de données collectées au cours de votre prise en charge (âge, sexe, durées d'hospitalisation) et éléments relatifs à l'activité des professionnels de santé étant intervenus à votre encontre (à partir du logiciel de codification/traçabilité des actes).

Les données de cette étude seront conservées pour une durée de 15 ans après la fin de l'étude.

Cette recherche est réalisée en collaboration avec le laboratoire Motricité, Interactions, Performance (UR 4334) de Nantes Université (Guillaume Le Sant, MK, PhD).

Votre participation à la recherche, au cas où vous donneriez votre accord, ne pourra vous être confirmée qu'à la condition que vous remplissiez tous les critères d'inclusion pour participer à cette recherche.

Si le nombre de personnes prévues dans l'étude a été atteint, vous pourriez finalement ne pas être inclus(e) dans l'étude bien qu'ayant signé un consentement. Dans ce cas précis, vous ne serez pas suivi(e) dans le cadre de l'étude et les données vous concernant le cas échéant seront détruits. Cela ne portera, en aucun cas, préjudice à la qualité de votre prise en charge médicale.

Cette recherche ne présente pas de risque pour votre santé. Les résultats qui en seront issus serviront à la recherche et n'apporteront pas d'informations supplémentaires concernant votre santé. Ils favoriseront le développement des connaissances dans le domaine de la prise en charge masso-kinésithérapique et sa place dans l'optimisation du parcours de soin patient.

TRAITEMENT DES DONNEES

Le traitement de vos données personnelles a pour fondement juridique l'intérêt public que revêt cette recherche (article 6.1.e du Règlement (UE) 2016/679 du Parlement européen et du Conseil du 27 avril 2016 relatif à la protection des personnes physiques à l'égard du traitement des données à caractère personnel et à la libre circulation de ces données (RGPD)). Il s'agit d'une des exceptions prévues par l'article 9 du même texte, permettant le traitement des données de santé.

Le traitement est conforme à la méthodologie de référence MR004 publiée par la CNIL.

Dans le cadre de cette recherche, un traitement informatique de vos données personnelles va être mis en œuvre : cela permettra d'analyser les résultats de la recherche et de remplir l'objectif de la recherche.

Pour cela, les données vous concernant seront recueillies dans un fichier informatique auquel aura accès le Promoteur de la recherche (CHU de Nantes).

Afin d'assurer leur confidentialité, vos données seront identifiées par un numéro de code et vos initiales.

Les personnes intervenant dans la recherche dans le cadre de leurs missions auront accès à vos données codées.

Les seules personnes qui auront accès à vos données directement identifiantes sont les personnes qui assurent votre prise en charge dans le cadre de la recherche et les personnes responsables du contrôle et de l'assurance qualité de la recherche.

Vos données pourront, dans des conditions assurant leur confidentialité, via des plateformes ou serveurs sécurisés, faire l'objet de transferts nationaux ou internationaux (comme, par exemple, aux personnes ou sociétés agissant pour le compte du CHU, aux autorités sanitaires habilitées) pour cette étude, ou pour d'autres recherches ultérieures, exclusivement à des fins scientifiques. Au sein de l'Europe la protection de vos données est garantie (Règlement européen UE 2016/679). Hors Europe vos données pourront être transmises dans des états n'ayant pas le même niveau d'exigence en termes de protection des données. Le cas échéant, le Promoteur prendra toutes les mesures nécessaires pour protéger les données recueillies et devra s'engager à assurer un niveau de sécurité équivalent à celui couvert par les lois françaises et européennes pour les données envoyées à l'étranger. Si vous le souhaitez, vous pouvez obtenir la copie de l'ensemble des mesures prises pour assurer l'ensemble de la protection de vos données auprès du Délégué à la Protection des Données (DPD) voir en fin de document, paragraphe « vos contacts ».

Vos données pseudonymisées (identifiées par un numéro de code et vos initiales) seront susceptibles d'être exploitées dans le cadre de publications ou de communications ; dans ce cas votre anonymat sera préservé.

Conformément aux dispositions de la loi relative à l'informatique aux fichiers et aux libertés (loi modifiée du 6 janvier 1978), et du Règlement (UE) 2016/679 du Parlement européen et du Conseil du 27 avril 2016 relatif à la protection des personnes physiques à l'égard du traitement des données à caractère personnel et à la libre circulation de ces données (RGPD), vous disposez d'un droit d'accès, de rectification, de limitation et d'opposition du traitement de vos données personnelles. Si vous décidez de retirer votre consentement pour participer à la recherche, les données obtenues avant que celui-ci n'ait été retiré seront utilisées conformément à l'article L.1122-1-1 du CSP. Les données recueillies après le retrait de votre consentement ne seront pas utilisées pour cette recherche et resteront destinées à l'usage strict du soin. Vous pouvez également porter une réclamation auprès d'une autorité de contrôle (CNIL pour la France : https://www.cnil.fr/fr/webform/adresser-une-plainte/).

Ces données pourront être utilisées lors de recherches ultérieures exclusivement à des fins scientifiques. Vous pouvez retirer votre consentement à cette utilisation ultérieure ou exercer votre faculté d'opposition à tout moment.

Vous pouvez également accéder directement ou par l'intermédiaire d'un médecin de votre choix à l'ensemble de vos données médicales en application des dispositions de l'article L 1111-7 du Code de la Santé Publique.

Vos données seront conservées tout au long de la recherche. Après la fin de la recherche, les données seront archivées pour une durée de 15 ans, puis détruites.

L'investigateur pourra vous informer, sur votre demande, des résultats globaux de cette recherche.

Pour en savoir plus ou exercer vos droits concernant vos données, voir en fin de document, paragraphe « vos contacts ».

Cette recherche est conforme :

- à la loi « Informatique et Libertés » du 6 janvier 1978 modifiée et la loi n° 2018-493 du 20 juin 2018 relative à la protection des données personnelles
- au Règlement (UE) 2016/679 du Parlement européen et du Conseil du 27 avril 2016 relatif à la protection des personnes physiques à l'égard du traitement des données à caractère personnel et à la libre circulation de ces données (RGPD)Ce projet ainsi que le présent document ont été présentés au Groupe Nantais d'éthique dans le domaine de la Santé GNEDS.

Votre participation à cette recherche est libre. Vous pouvez refuser de participer à cette recherche, et vous pouvez à tout moment vous retirer de cette recherche, sans préjudice. Cela n'aura aucune conséquence sur la qualité des soins qui vous seront donnés; vous devez simplement en informer l'investigateur.

VOS CONTACTS :

Pour toute question concernant l'étude, retrait de consentement, ou pour exercer vos droits concernant vos données (accès, rectification, etc…), votre contact privilégié est :

L'investigateur coordonnateur de la recherche :

BLANDIN Marie

✉ marie.blandin@chu-nantes.fr

☎ 02.40.08.33.33

Pour toute question générale sur le traitement de vos données :

Le promoteur de la recherche, responsable du traitement :

CHU de Nantes, direction de la recherche et de l'innovation

5 allée de l'Ile Gloriette, 44093 NANTES Cedex 1

Le Délégué à la Protection des Données (DPD) / Data Protection Officer (DPO) :

vosdonneespersonnelles@chu-nantes.fr

Merci de conserver cette notice d'information
